# Establishment and bioinformatics analysis of a four-miRNA prognostic signature for pleural mesothelioma

**DOI:** 10.7150/jca.101914

**Published:** 2024-10-21

**Authors:** Xi Yang, Yaru Lin, Baoru Dong, Bin Li, Ruai Liu, Xinmeng Wang, Jinsong Li, Xue Cheng, Zhengliang Li, Wei Xiong

**Affiliations:** 1 Department of Biochemistry and Molecular Biology, College of Basic Medical Sciences, Dali University, Dali 671000, Yunnan, China.; 2 Yunnan Provincial Key Laboratory of Entomological Biopharmaceutical R&D, College of Pharmacy, Dali University, Dali 671000, Yunnan, China.; 3 Department of General Surgery, The Second People's Hospital of Bao Shan, Bao Shan 678000, Yunnan, China.; 4 Department of Radiology, The First Affiliated Hospital of Dali University, Dali University, Dali 671000, Yunnan, China.

**Keywords:** Pleural mesothelioma (PM), Prognostic signature, Biomarker, Bioinformatics, The Cancer Genome Atlas (TCGA), Gene Expression Omnibus (GEO)

## Abstract

**Objective:** Pleural mesothelioma (PM), an uncommon yet highly aggressive malignant neoplasm, has a very poor prognosis with a median survival of less than one year after diagnosis, morbidity and mortality due to PM are on the rise year by year worldwide. Our research aims to utilize molecular characteristics and microRNAs (miRNAs) as a breakthrough in predicting the survival of PM patients, hoping to find a molecular mechanism that can predict the survival of PM patients.

**Methods:** The miRNA expression profiles and corresponding clinical information of patients with PM were obtained from The Cancer Genome Atlas (TCGA) database, a miRNA-based prognostic signature was developed using Cox regression analysis in the training cohort, which was validated in the testing cohort and complete cohort. The association between miRNA levels and survival outcomes was determined, the miRNAs in prognostic model were experimentally validated by quantitative real-time PCR (qRT-PCR) in cell lines. Target genes of prognostic miRNAs were identified using TargetScan, miRDB, and miRTarBase databases, biological function prediction of which was accomplished by GO and KEGG analysis. Gene Expression Omnibus (GEO) database was utilized for core targets recognition, immune infiltration and survival analysis were conducted to investigate the relationship between core targets and immune cells by bioinformatics analysis.

**Results:** This miRNA-related prognostic risk model can effectively stratify patients into high-risk and low-risk groups, and have good sensitivity and specificity to assess the prognosis of patients with PM, which can also be used as an independent prognostic factor for overall survival (OS) prediction in patients with PM, the OS for patients in high-risk group was significantly poorer compared with patients in low-risk group. Moreover, all four miRNAs (hsa-miR-181a-2-3p, hsa-miR-491-5P, hsa-miR-503-5p, and hsa-miR-3934-5p) were found to be differentially expressed in PM cell lines as compared with normal cell line, GO and KEGG analysis revealed that target genes of miRNAs in prognostic model were involved in multiple tumor-associated signaling pathways and functions in PM, core miRNA targets also correlated with immune cell infiltration, indicating their potential role in PM initiation and progression.

**Conclusions:** A robust four-miRNA prognostic signature with great performances in prediction of the OS for PM patients was developed in our study, providing new avenues for the prognostic predication of PM.

## Introduction

Pleural mesothelioma (PM) was first proposed in 1947 and further described as "endothelioma of the pleura" in 1955^1^, ^2^. It is a highly aggressive cancer with poor prognosis, which is associated with asbestos exposure, the latency period between start of exposure and clinical diagnosis is over 40 years^3^. Due to its aggressive nature, the median survival time was less than 2 years after diagnosis^4^. The incidence of malignant mesothelioma in Australia is among the highest in the world as a result of widespread use of asbestos by industry and in construction throughout the 20th century, with around 700 to 800 patients diagnosed each year, of which 94% constitute PM, and the remaining 6% are mostly peritoneal mesothelioma^5^, ^6^.

PM has heterogeneous histological features and can be classified into three main histological subtypes, namely epithelioid, sarcomatous, and biphasic, patients with the sarcomatoid subtype are considered to have the worst prognosis^7^, ^8^. The incidence of PM is increasing steadily around the world, morbidity and mortality of PM varies greatly in different regions. It is estimated that the incidence peak in Western Europe and North America will be reached around 2020, and in most of Africa, Eastern Europe, South America and Asia, asbestos production and commercial use are uncontrolled, and may continue to rise in coming decades^4^, which means that PM will remain a major global health issue even after the peak of incidence rate. Despite recent advances in research, the molecular basis of PM is still largely unknown, which has become the most critical limitation in developing effective PM targeting strategies^9^. Patients with PM are always diagnosed in late stage, current available treatments cannot be implemented in time, highlighting the need for identifying suitable prognosis biomarker that reveal the mechanisms underlying tumorigenesis for personalized prognosis prediction and aid in the development of effective treatment strategies, to enhance therapeutic outcomes and improve the management.

Recent researches have shown that miRNAs regulate various steps in carcinogenesis, including cancer initiation, progression, metastasis, and patient survival^10^, they are a group of small non-coding RNAs that post-transcriptionally control expression of genes in a sequence-specific manner by targeting mRNAs^11^. It has been reported that miRNAs are stably expressed in tissues and diverse biological fluids^12^, ^13^, pointing to its potential as prognostic indicators of patients with PM. MiRNA expression profiling were aberrant in several human cancers, and showing promise in defining malignant status in retrospective studies^14^, based on its measurable characteristics of physiological and pathological conditions, miRNAs can be used for the prognosis, diagnosis and treatment effect evaluation of cancer, including PM. Several miRNAs have been identified related to PM prognosis, for example, there is evidence that miRNA-29 was an independent prognostic factor for time to progression as well as survival after surgical cytoreduction^15^, high expression of miR-98 in tissues was demonstrated to be significantly associated with poor prognosis of asbestos-exposed PM patients^16^. As well, high expression of miR-137 was linked to poor patient survival, and increased miR-137 levels inhibited the growth and colony formation of PM cells^17^. However, most studies only focused on the identification of a single miRNA marker, which does not necessarily produce reliable predictive results.

In this study, a novel four-miRNA signature that can effectively predict overall survival (OS) was developed and validated through the univariate and multivariate Cox regression analyses using the RNA sequencing data in The Cancer Genome Atlas (TCGA) database (https://portal.gdc.cancer.gov/), quantita-tive reverse transcription-based polymerase chain reaction (qRT-PCR) was used to detect the miRNAs expressions in mesothelioma cell lines, the possible mechanisms of these miRNAs were investigated by following bioinformatics analysis. Overall, these findings were of promising value to the prognostic prediction of patients with PM, along with the development of immune-related treatment strategy.

## Materials and Methods

### Data acquisition and pre-processing

The RNA sequencing data and corresponding clinical follow-up information obtained from PM patient samples (TCGA-MESO) were downloaded from the publicly available TCGA database, a total of 84 miRNA expression profiles were extracted after removing cases that missing survival time and status, and remaining cases were applied for further survival analysis. The raw data in the dataset were annotated to obtain the miRNA expression levels, miRNAs with expression levels less than or equal to 1 were filtered out, and the average expression values of same miRNAs were considered the expression values of the corresponding miRNAs. Standardization of the level of miRNA expression for each sample was normalized with the edgeR package in R language.

### Identification of miRNAs associated with overall survival

Survival analysis is an inference to study the survival time and outcome, as well as the relationship and extent between the numbers of influencing factors^18^. The “caret” R package was used to randomly and equally group the TCGA-MESO dataset into training dataset and testing dataset, correlation of miRNA with OS was evaluated by “survival” R package, miRNAs related to prognosis were filtered with expressing significance *p* values<0.001 through univariate Cox regression analysis in the training set.

### Development and validation of the four-miRNA prognostic signature

Each of above prognostic related miRNA identified in the training cohort utilizing univariate Cox regression was included in the multivariate Cox regression in order to construct the miRNA signature of PM. To evaluate the robustness and reliability of the prognostic signature, subsequently, this four-miRNA signature was validated in the testing cohort and complete cohort. For each independent cohort, the risk score of patients with PM was calculated using the coeffcients derived from the training dataset, based on the median risk score, patients were divided into a high-risk and a low-risk group. Receiver operating characteristic (ROC) curve analyses were generated for survival predictions to evaluate the specificity and sensitivity of the miRNA signature and to determine if the innovative model might effectively anticipate the survival of patients with PM. The Kaplan-Meier approach was utilized to examine the survival rate between the two risk groups, whereas the log-rank test was utilized to compare the outcomes. Furthermore, we determined the distribution of risk scores, the survival status of patients in different risk categories and the expression characteristics of the four miRNAs. The independent prognostic factor analysis between the risk model and clinical characteristics was assessed using univariate and multivariate analysis, survival analysis was employed to determine whether each miRNA in risk prognosis model was substantially correlated with survival rate.

### Cell culture

The human mesothelioma cell lines (NCI-H2452, NCI-H28) and normal mesothelial cell line (MeT-5A) used in this study were purchased from Pricella biotech co., ltd. (Wuhan, China) and Cellcook biotech co., ltd. (Guangzhou, China). All cell lines were cultured in RPMI-1640 medium (Gibco) supplemented with 10% fetal bovine serum (Gibco) and 1% penicillin-streptomycin (Invitrogen) at 37°C in a humidified incubator containing 5% carbon dioxide. All cells were authenticated via short tandem repeat (STR) profiling. Mycoplasma status was checked often using the TransDetect Luciferase Mycoplasma Detection Kit (TransGen Biotech, Beijing, China). The medium was changed every 2~3 days, and 0.25% trypsin (Thermo Fisher HyClone, Utah, USA) was added for digestion and passage when the cells were 80% confluent.

### The quantitative real-time PCR verification of molecules in the prognostic model

Total RNA and miRNAs were isolated from the cell lines by using the miRcute miRNA Isolation Kit (CAT No. DP501, TIANGEN), the miRNA reverse transcription was performed using miRcute Plus miRNA First-Strand cDNA Kit according to the manufacturer's instructions (CAT No. KR211, TIANGEN), Quantitative reverse-transcription real-time PCR (qRT-PCR) was performed using miRcute Plus miRNA PreMix (CAT No. FP411, TIANGEN) on an ABI 7500 Real-Time PCR system (Applied Biosystems, Waltham, MA, USA). The U6 snRNA (ID001973, Thermo Fisher Scientific, Waltham, MA, USA) was used as an internal reference, and the relative expression levels of the miRNAs were calculated with the comparative threshold cycle (2^-ΔΔCt^) method. The experiment was performed in triplicates. The following primers were used: hsa-miR-181a-2-3p FW 5′-GCACCACTGACCGTTGACTGTACC-3′; hsa-miR-491-5p FW 5′-GTGGGGAACCCTTCCATGAGG-3′; hsa-miR-503-5p FW 5′-GCAGCGGGAACAGTTCTGCAG-3′; hsa-miR-3934-5p FW 5′-GTTCAGGTGTGGAAACTGAGGCAG-3′; U6 snRNA FW 5′-ATGGACTATCATATGCTTACCGAT-3′.

### Prediction of miRNA targets and construction of the miRNA-mRNA regulatory network

Targets of the four miRNAs in the prognostic model were predicted by three online databases, including TargetScan (https://www.targetscan.org), miRDB (http://www.mirdb.org), and miRTarBase (https://mirtarbase.cuhk.edu.cn). The targets, which were simultaneously predicted and overlapped in three databases, were selected for further study. According to the regulatory relationship between miRNA and mRNA, the miRNA-mRNA relationship pair is clarified, and the miRNA-mRNA regulatory network was established and visualized by Cytoscape version 3.9.1 software using the four miRNAs and overlapping target genes.

### MiRNA targets correlated biological pathways and processes

GO function enrichment analysis including biological process (BP), cellular component (CC), and molecular function (MF) and KEGG pathway analysis of candidate target genes were implemented using the R package “ClusterProfiler”. The GO terms and KEGG pathways with a P-value of <0.05 were considered significantly enriched function annotations. Column chart was plotted using R software.

### Identification of core miRNA targets

In order to increase prediction reliability of downstream target genes, here, we conducted our data mining analysis for PM by taking the intersection of differentially expressed genes acquired from Gene Expression Omnibus (GEO) database (https://www.ncbi.nlm.nih.gov/geo/) by comparing tumor tissues versus normal tissues (GSE2549, GSE42977 and GSE51024) and selected miRNA targets which predicted in above three databases as the result of core miRNA targets, and retain targets that had the opposite expression trend with miRNAs, implying that they may have direct targeting relationships.

### Correlation analysis between core targets and immune cell infiltration in PM

To better understand whether there is a relationship between the core targets of the prognostic model-related miRNA and the infiltration of immune cells in PM, immune cell infiltration analysis of key targets was performed using the gene module of the TIMER2.0 database (http://timer.cistrome.org/). The plots showing relationship between infiltration estimates and gene expression levels were visualized and *p*<0.05 with Rho>0.3 was considered statistically significant.

### Association between survival and different fractions of immune cells

The outcome module of the TIMER2.0 database was used to demonstrate the relationship between clinical outcomes and immune cell infiltration, with the flexibility to explore the effect of infiltration level of immune cells on the survival of PM patients.

### Association between core targets and immune cell population

CIBERSORT is a method of analyzing the composition and abundance of immune cells in a mixed-cell population using gene expression data based on the principle of linear support vector regression^19^. All samples of PM in TCGA database were divided into high-expression group and low-expression group according to VPS37B expression levels, the gene expression matrix was uploaded to CIBERSORT and derived a matrix of 22 types of immune cells. CIBERSORT *p* <0.05 was used to filter the samples. The distribution of 22 types of immune cells in the samples was calculated, and the difference in immune cells between the two groups was displayed in a violin plot using the ggplot2 package in R language.

### Statistical analysis

R programming software (version 4.1.2) was used in statistical analyses. To compare the differences in the survival of patients between the two risk groups, the Kaplan-Meier analysis combined with the log-rank test from the “survival” R package was utilized. Multivariate and univariate analyses were performed utilizing the Cox proportional hazards regression (HR) model and corresponding 95% confidence intervals (CIs) to determine the prognostic significance of the four-miRNA signature. Data for various clinicopathological factors were analyzed using *chi-square* test. Independent *t*-tests were performed to compare continuous variables between the two groups. All statistical tests were two-sided and *p*<0.05 was considered significantly different.

## Results

### Construction of the risk assessment model

In order to determine the miRNAs associated with prognosis of patients with PM, a total of 84 PM clinical samples with miRNA expression data were obtained. Patients were randomly divided into training set and test set by using createDataPartition function in caret package, with the training set containing 44 samples and the validation set containing 40 samples. The "survival" R package was utilized to perform univariate Cox proportional hazards regression analysis on each miRNA in training set, and seven miRNAs were statistically significantly correlated with prognosis while a *P*-value of <0.001, the results were shown in table 1. Next, in order to construct the miRNA signature, multivariate Cox regression analysis was employed on the seven miRNAs, four miRNAs were selected as signature miRNAs that can optimally predict the OS of patients with PM, and the results of multivariate Cox regression analysis are shown in table 2. According to the regression coefficients and expressions of four miRNAs, the risk score formula of 4-miRNA signature was computed as follows:

Riskscore=(hsa-miR-181a-2-3p)×0.563118-(hsa-miR-491-5p)×0.49509+(hsa-miR-503-5p) ×0.32936-(hsa-miR-3934-5p)×0.38379

### Validation of the four-miRNA signature

**Figure 1:**
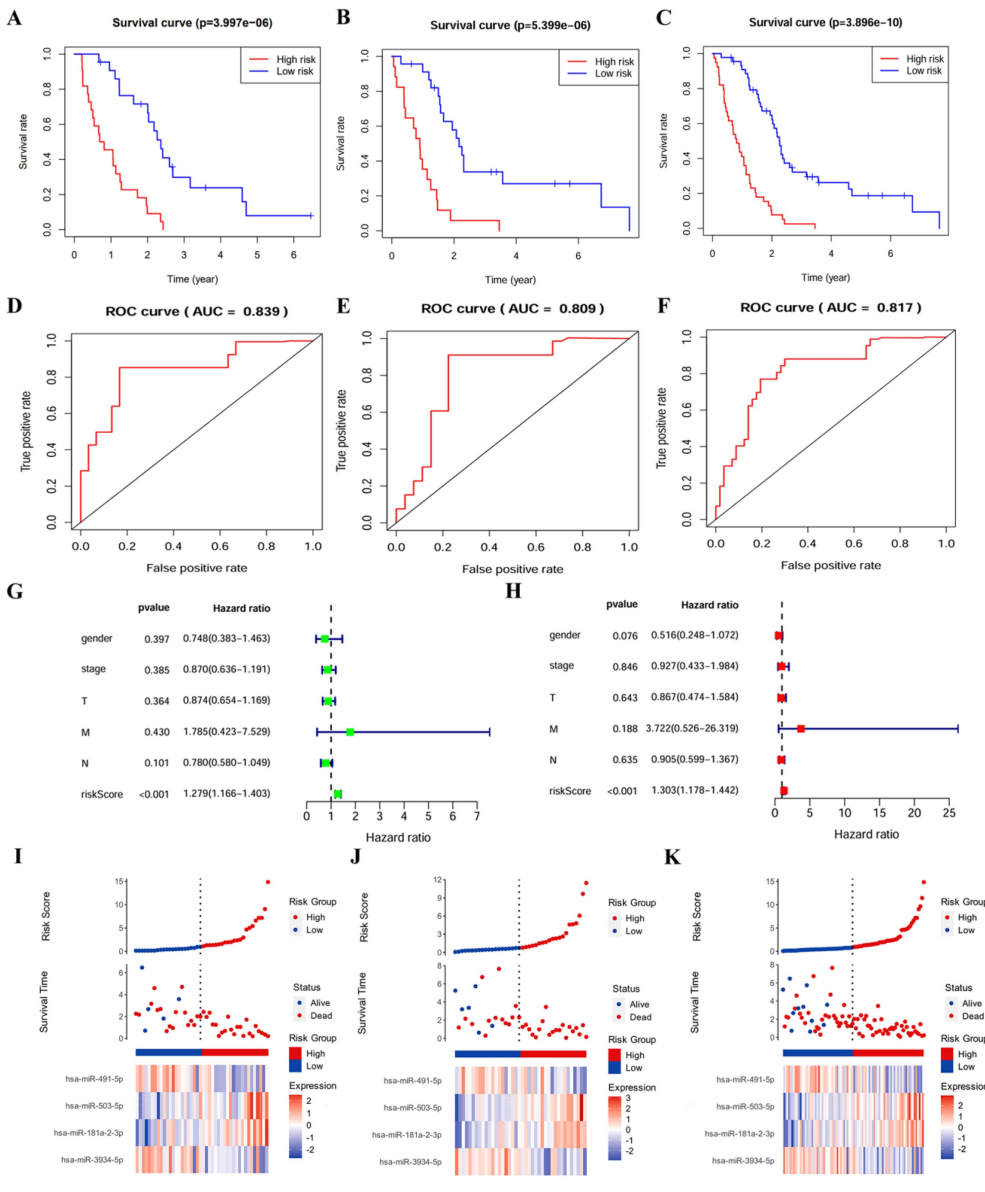
Validation of the four-miRNA signature. **(A)** Kaplan-Meier curve in training cohort. **(B)** Kaplan-Meier curve in testing cohort. **(C)** Kaplan-Meier curve in complete cohort. **(D)** ROC curve in training cohort. **(E)** ROC curve in testing cohort. **(F)** ROC curve in complete cohort. **(G)** Forest map of univariable analysis for risk score and clinic-pathological features. **(H)** Forest map of multivariable analysis for risk score and clinic-pathological features. **(I)** Distribution of risk score, survival status, and miRNA expression in training cohort. **(J)** Distribution of risk score, survival status, and miRNA expression in testing cohort. **(K)** Distribution of risk score, survival status, and miRNA expression in complete cohort.

To evaluate the robustness and effectiveness of the prognostic model, we validated the four-miRNA signature in the testing cohort and complete cohort. Patients were divided into low-risk groups and high-risk groups based on median risk score. According to K-M survival analysis, the survival rates for patients in high-risk group were significantly poorer compared with patients in low-risk group (*p* < 0.001) (Figure 1A and B and C). The AUC values obtained from ROC curve analysis in all three sets were more than 0.8 (Figure 1D and E and F), confirming the sensitivity and specificity of this model and survival prediction accuracy. The prognosis of cancer patients is affected by a variety of complex clinical factors, univariate and multivariate independent prognostic factor analysis demonstrated that risk score could be a significant independent predictive predictor of PM patients (*p* <0.001) (Figure 1G and H). In addition, the risk score of patients was negatively associated with survival. Furthermore, the risk heatmap revealed that the levels of hsa-miR-181a-2-3p and hsa-miR-503-5p correlated positively with the risk scores, while the levels of hsa-miR-491-5p and hsa-miR-3934-5p correlated negatively with the risk scores (Figure 1I and J and K).

### Survival analysis of miRNAs in the prognostic model

**Figure 2:**
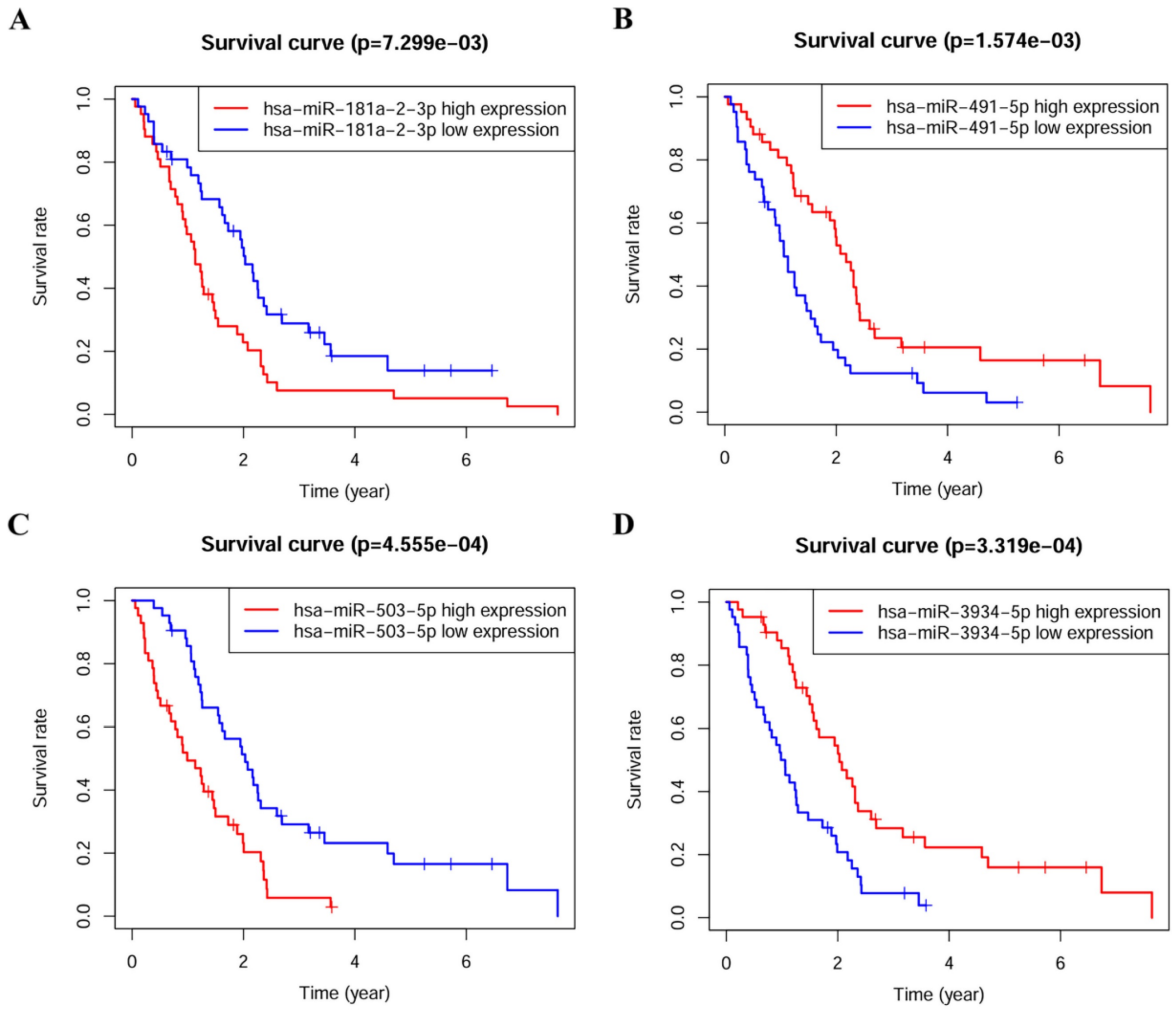
Kaplan-Meier survival analysis of four prognostic-related miRNAs. **(A)** hsa-miR-181a-2-3p.**(B)** hsa-miR-491-5p.**(C)** hsa-miR-503-5p.**(D)** hsa-miR-3934-5p.

The association between miRNA levels with survival rates of PM patients was analyzed separately, patients were divided into high and low expression groups according to the median expression values of miRNAs, a highly-significant difference was found in survival rates between high and low expression groups, and all of them were statistically significant (*p* < 0.05) (Figure 2).

### Experimental verification of miRNAs

**Figure 3:**
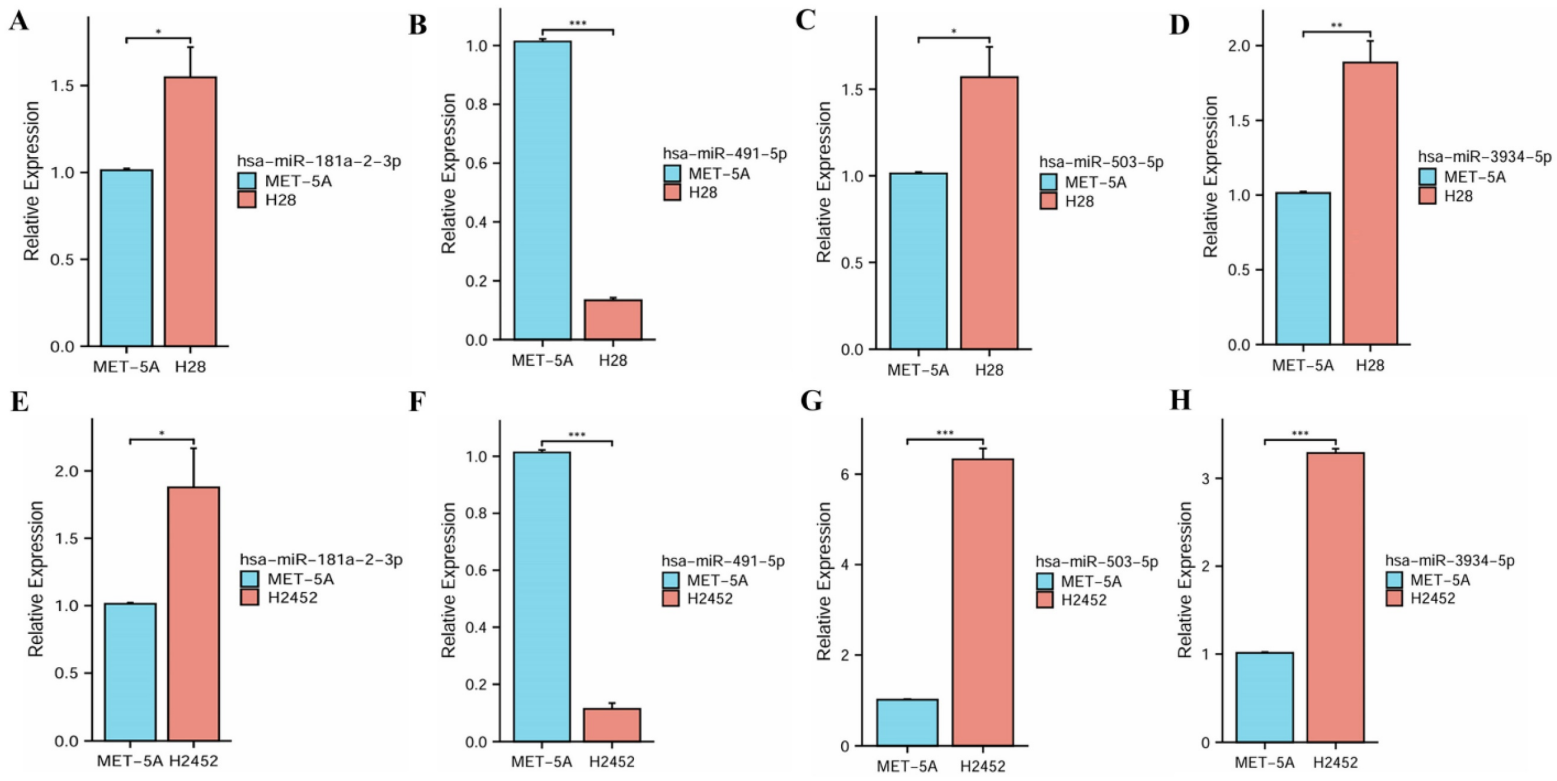
The expression levels of four miRNAs between normal mesothelial cell line and PM cell lines. **(A, E)** hsa-miR-181a-2-3p. **(B, F)** hsa-miR-491-5p. **(C, G)** hsa-miR-503-5p. **(D, H)** hsa-miR-3934-5p.

The qRT-PCR assay was used to analyze the expressions of four miRNAs in human normal mesothelial cell line MeT-5A and PM cell lines NCI-H28 and NCI-H2452. These experimental results indicated that miR-181a-2-3p, hsa-miR-503-5p and hsa-miR-3934-5p had significantly high expressions in PM cell lines, while hsa-miR-491-5p had a significantly low expression, suggesting their roles in the progression of PM (Figure 3).

### Prediction of miRNA targets

**Figure 4:**
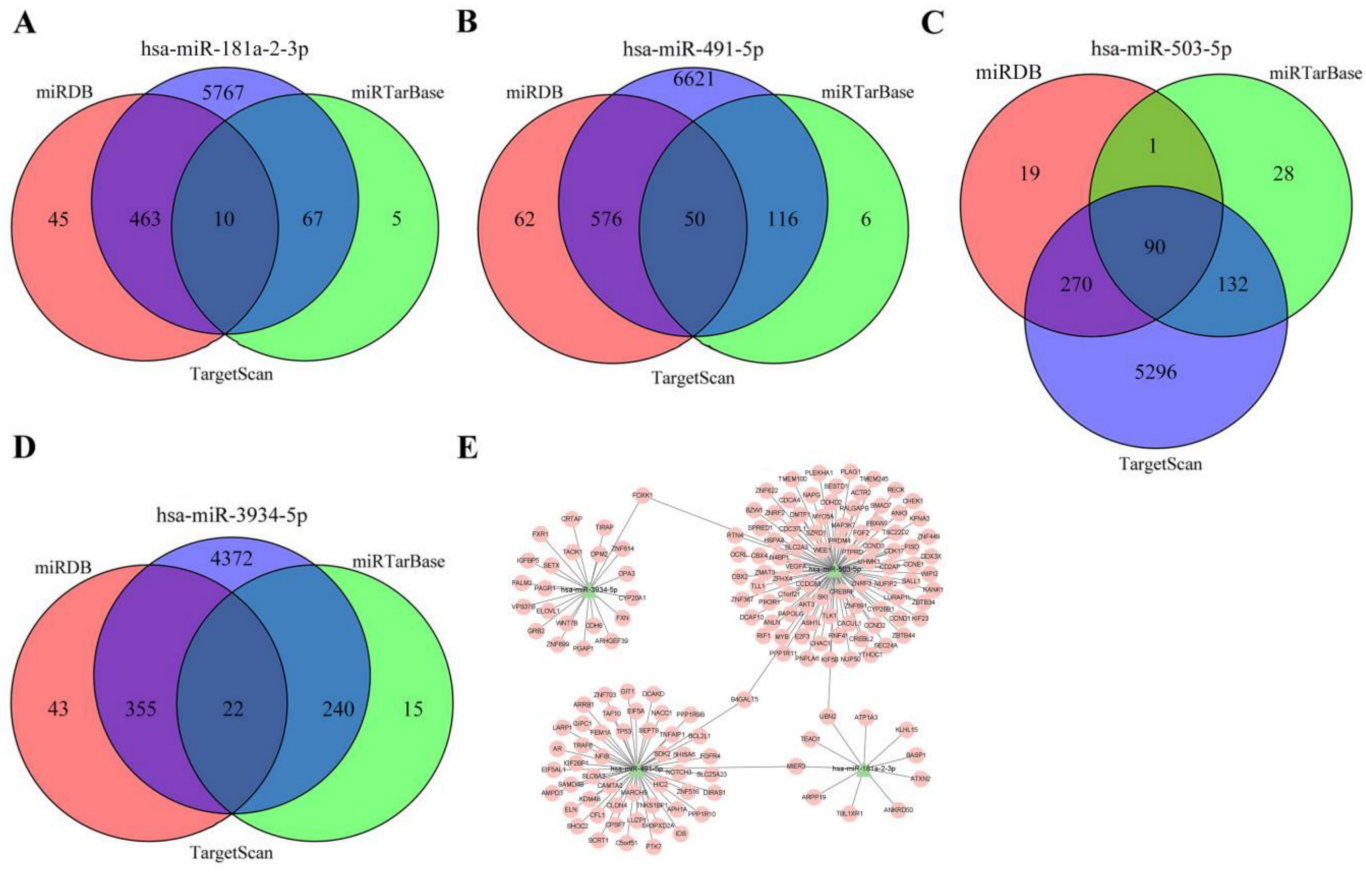
Target genes prediction. **(A)** Target genes of hsa-miR-181a-2-3p.**(B)** Target genes of hsa-miR-491-5p.**(C)** Target genes of hsa-miR-503-5p.**(D)** Target genes of hsa-miR-3934-5p. **(E)** miRNA-protein interaction network.

In order to explore the downstream regulatory mechanism of miRNAs in the prognostic model, TargetScan, miRTarBase, and miRDB databases were used to predict the downstream targets of four miRNAs. The mRNAs that intersected among three databases were finally identified as miRNA targets (Figure 4A and B and C and D). According to the relationship between miRNA and mRNA, cytoscape software was used to visualize the regulatory network, 172 miRNA-mRNA relationship pairs were finally screened out, one kind of miRNA may have several targets, and several kinds of miRNAs may act on one target, suggesting complex interactions and mechanisms between miRNAs and target genes lead to pathological states (Figure 4E).

### GO terms and KEGG pathways analysis

**Figure 5:**
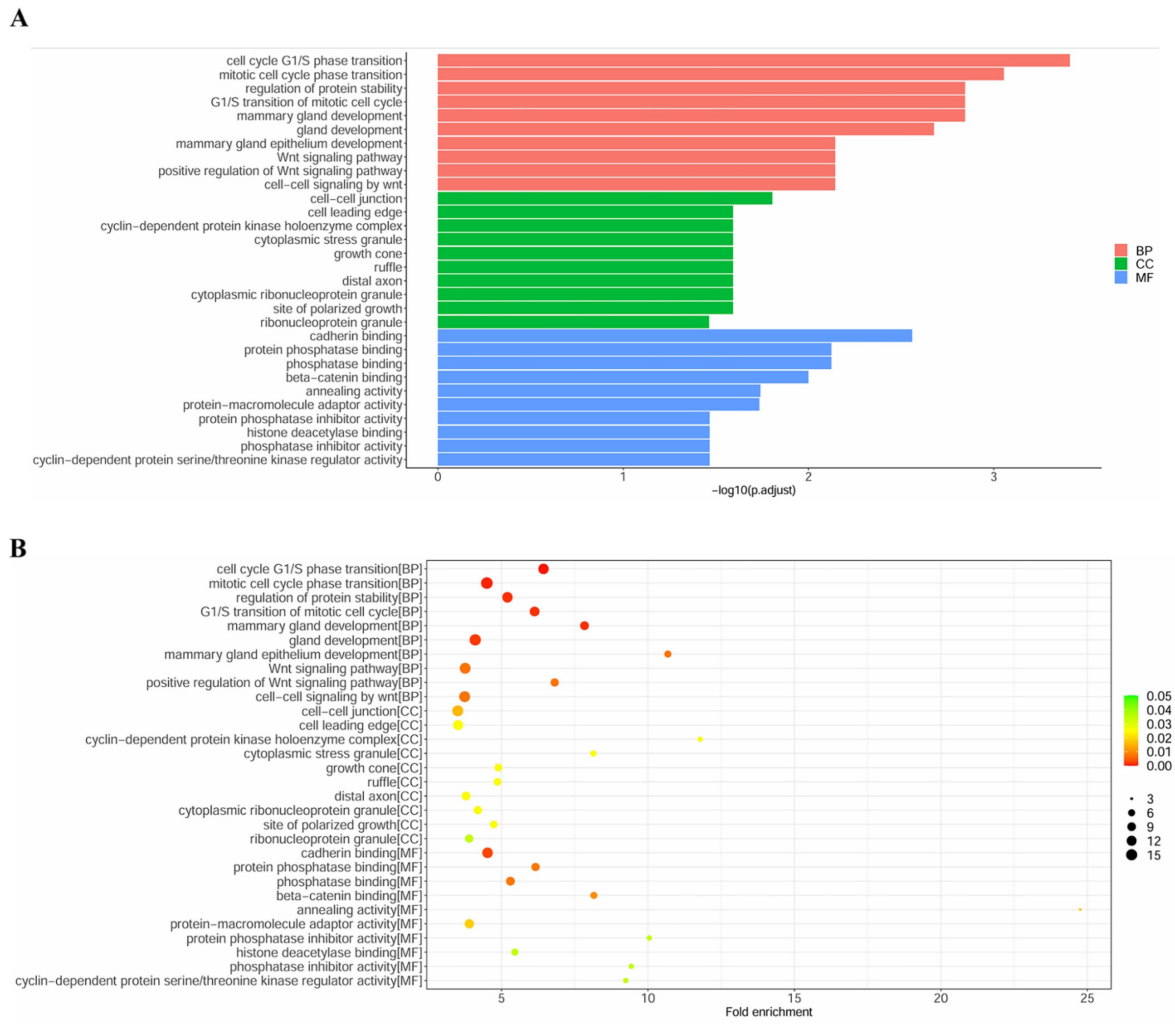
GO enrichment analysis of the target genes. **(A)** column plot. **(B)**scatter plot.

**Figure 6:**
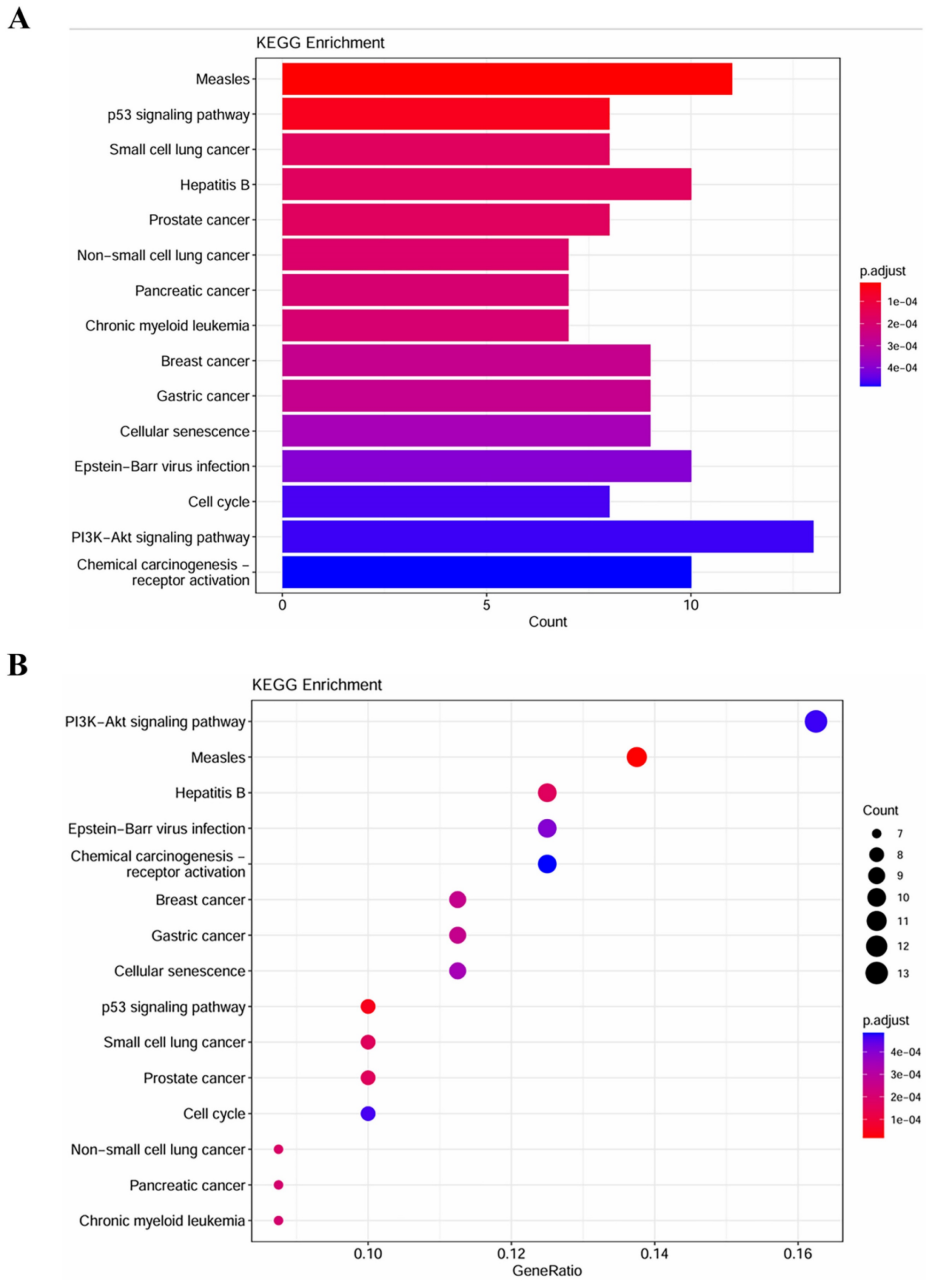
KEGG pathway enrichment analysis of the target genes. **(A)** column plot. **(B)**scatter plot.

To explore the biological function of miRNA targets in PM development, GO enrichment analysis and KEGG analysis were performed using R language. The results of GO enrichment analysis showed that target genes are involved in cell cycle G1/S phase transition, cell-cell junction, cadherin binding, etc (Figure 5). KEGG analysis showed that miRNA targets are involved in several cancer-related pathways, including small cell lung cancer, prostate cancer, pancreatic cancer, chronic myeloid leukemia, breast cancer, p53 signaling pathway, PI3K-Akt signaling pathway, etc (Figure 6).

### Identification of core miRNA targets

The expression values of genes in GSE2549, GSE42977 and GSE51024 datasets were normalized using the limma package in R software (Supplementary Figure 1A-F), differentially expressed genes were selected according to |log_2_FC|>1 and false discovery rate (FDR)≤0.05 (Supplementary Figure 1G-I). Three common genes (KIF23, TMEM100 and VPS37B) were screened (Supplementary Figure 1J), results of survival analysis for three genes showed that high expression of KIF23 and VPS37B independently predicted short OS in PM (Logrank *p* <0.05) (Supplementary Figure 1K and L), and these two genes were selected as core targets for subsequent analysis.

### Immune cell infiltration and immune cell survival analysis

**Figure 7:**
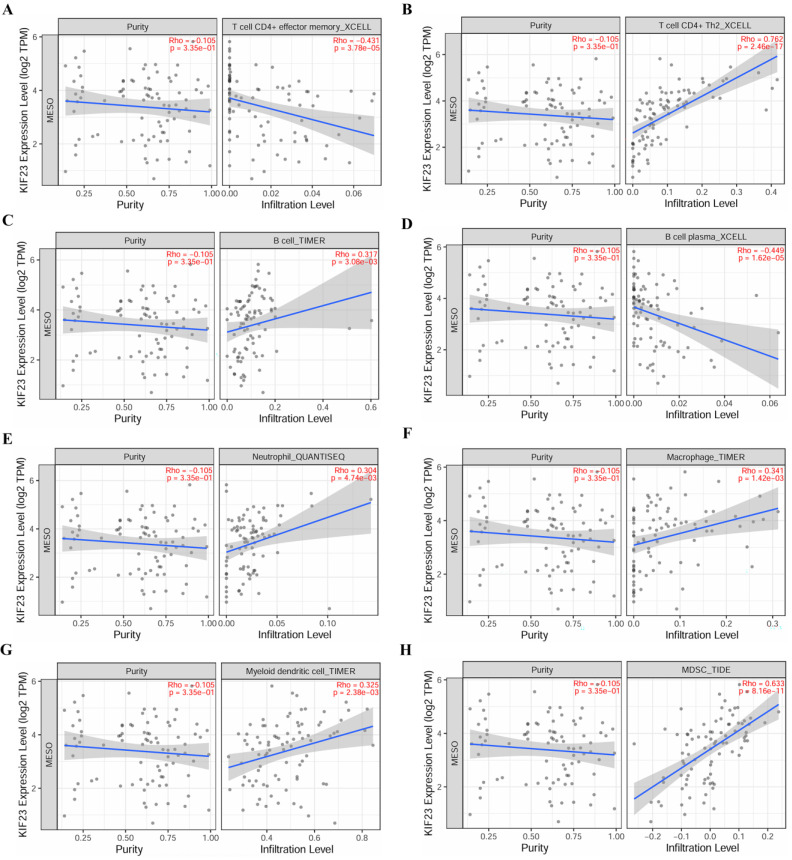
Correlation of KIF23 expression and immune cell infiltration. **(A)** T cell CD4^+^ effector memory. **(B)** T cell CD4^+^ Th2. **(C)** B cell. **(D)** B cell plasma. **(E)** Neutrophil. **(F)** Macrophage. **(G)** Myeloid dendritic cell. **(H)** MDSC.

**Figure 8:**
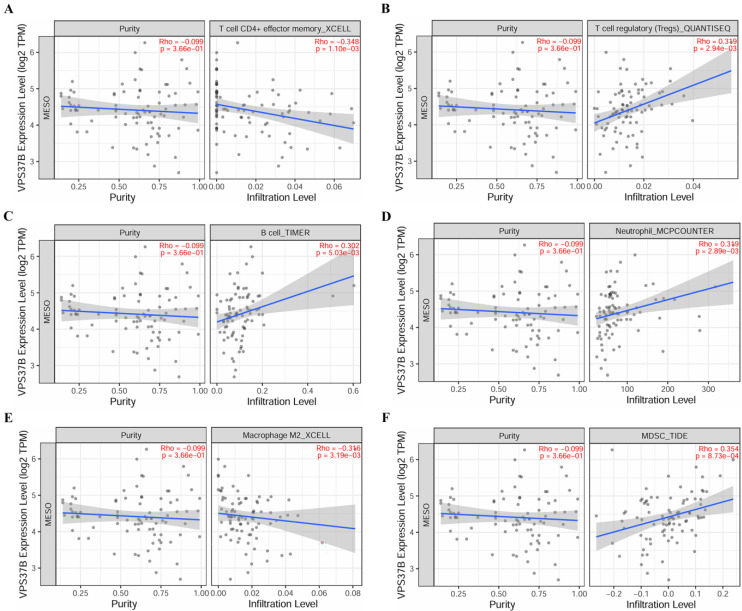
Correlation of VPS37B expression and immune cell infiltration. **(A)** T cell CD4+ effector memory. **(B)** T cell regulatory. **(C)** B cell. **(D)** Neutrophil. **(E)** Macrophage M2. **(F)** MDSC.

**Figure 9:**
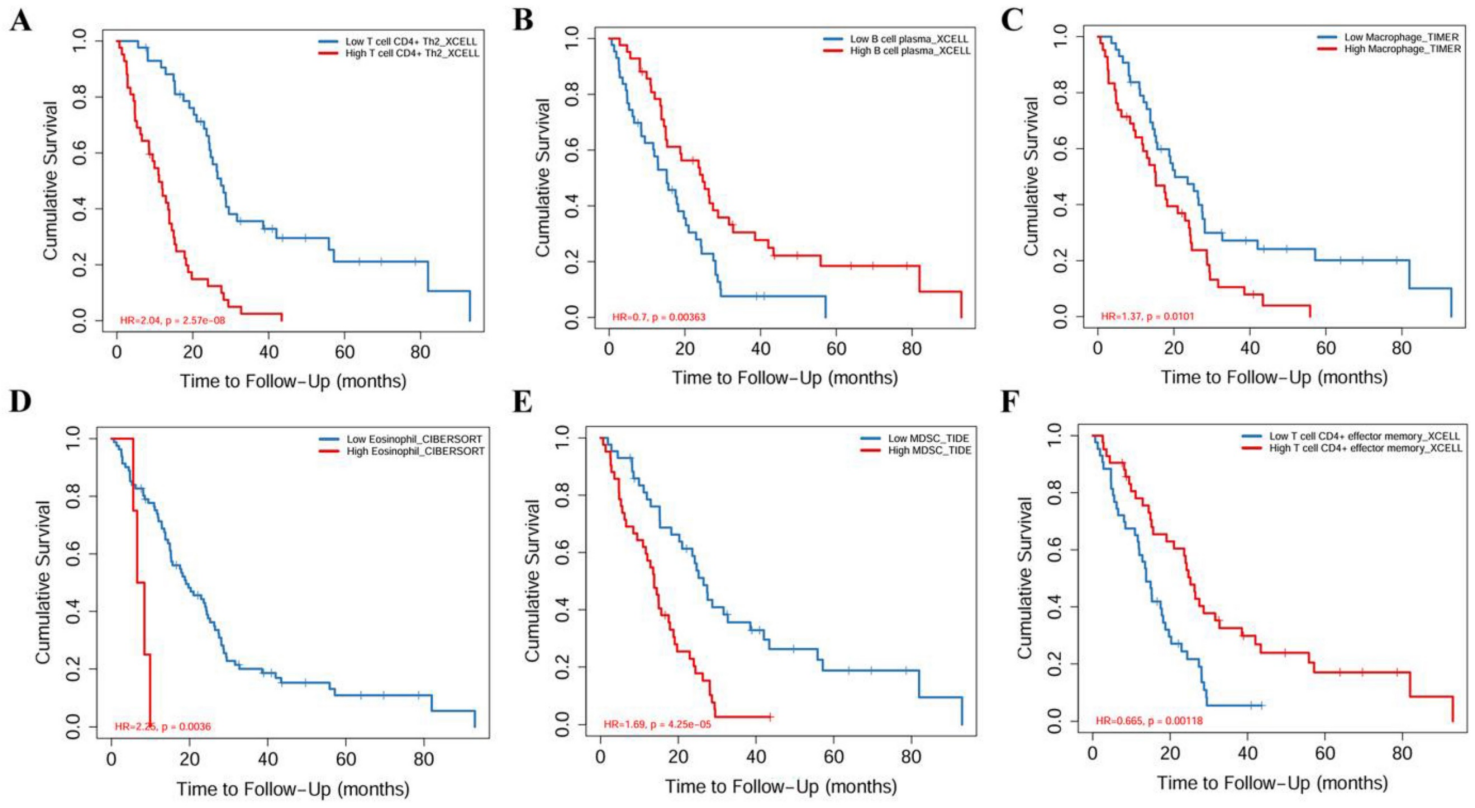
Kaplan-Meier survival analysis of immune cell infiltration. **(A)** T cell CD4+ Th2. **(B)** B cell plasma.** (C)** Macrophage. **(D)** Eosinophil. **(E)** MDSC. **(F)** T cell CD4+ effector memory.

The relationship between core miRNA targets expression and immune infiltration was explored by searching the TIMER2.0 database, immune cells with Rho value greater than 0.3 were visualized. KIF23 was significantly associated with infiltration of T cell CD4+ effector memory, T cell CD4+ Th2, B cell plasma and MDSCs (*p* <0.001), especially T cell CD4+ Th2 and MDSCs (Rho= 0.762, 0.633) (Figure 7). VPS37B was significantly associated with infiltration of T cell CD4+ effector memory, T cell regulatory (Tregs), B cells, neutrophils, M2 macrophages and MDSCs (*p* <0.01, Rho=-0.348, 0.319, 0.302, 0.319, -0.316, 0.354) (Figure 8). The results highlight the link between these genes and tumor immune cell infiltration, particularly with T cell CD4+ effector memory, B cells, neutrophils, macrophages, and MDSCs. Next, we used the outcome module of the TIMER2.0 database to explore the impact of infiltration levels of immune cells on survival outcomes in patients with PM. The results showed that T cell CD4+ Th2, B cell plasma, macrophages, eosinophils, T cell CD4+ effector memory and MDSCs infiltration levels have significant effects on survival outcomes (*p* <0.05) (Figure 9). In summary, these genes may be involved in the regulation of tumor immune microenvironment by influencing multiple immune cells, further affecting the prognosis of PM patients.

### Association analysis of core targets with immune cell population

**Figure 10:**
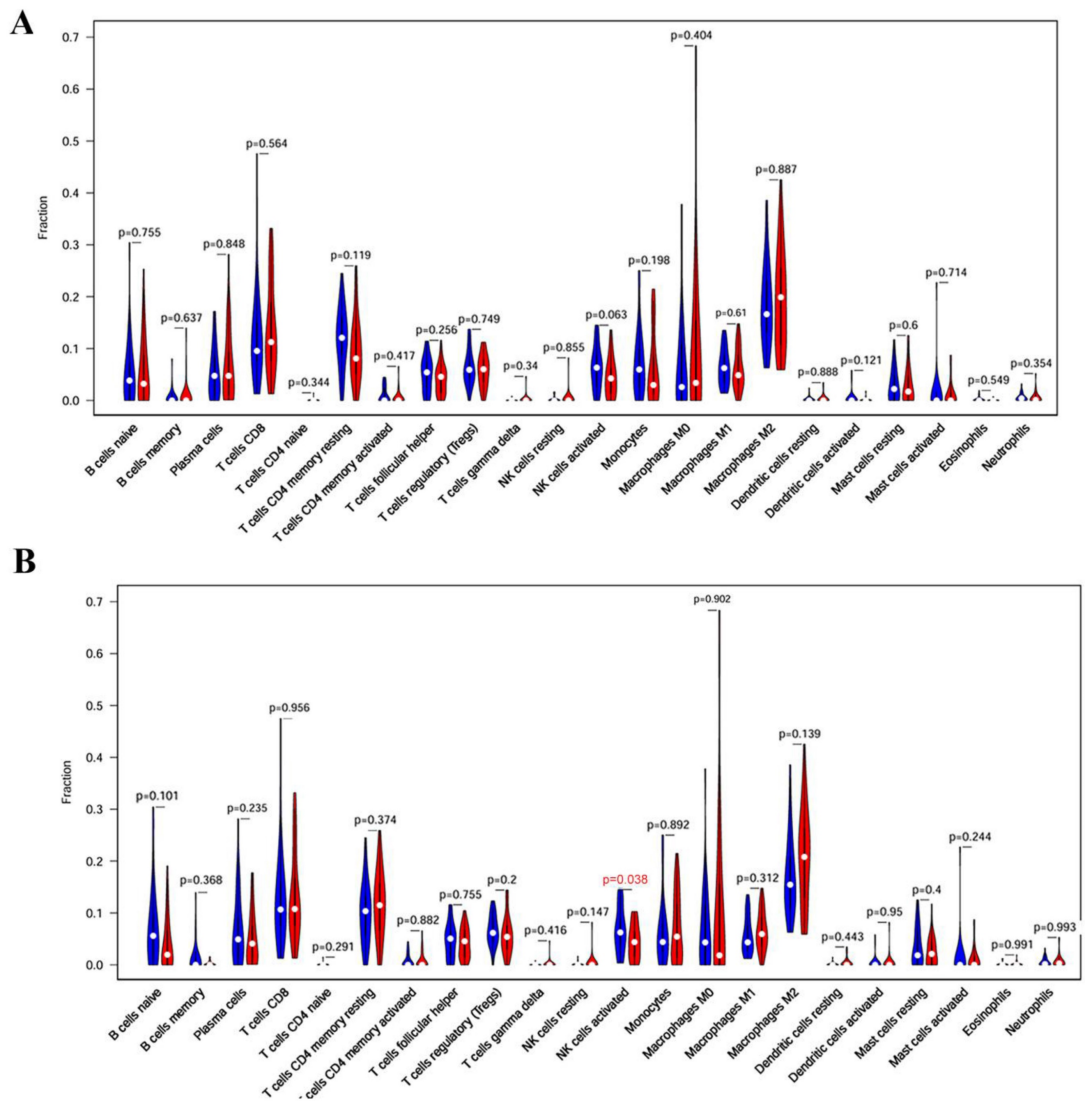
Association between core targets and immune cell population. **(A)** Immune cell populations in KIF23 high- and low-expression groups. **(B)** Immune cell populations in VPS37B high- and low-expression groups. (red: high expression group; blue: low expression group)

We further analyzed the relationship between core target genes and immune cell population. The results showed that the activated NK cells were significantly reduced in VPS37B-high expression group (*p* <0.05), indicating that VPS37B may affect the activity of NK cells, thus further affecting the development of PM (Figure 10).

## Discussion

PM is a rare cancer of the mesothelial cells, patients with PM had a very poor prognosis due to its long latency and non-specific symptoms^20^. Asbestos exposure is a major risk factor, causing high mortality rate of patients with PM^21^, however, a small proportion of PM patients do not appear to be associated with asbestos exposure, recent studies on PM oncogenesis have highlighted the role of tumor-related genes that can influence the development and prognosis of PM^22^. At present, many studies have focused on the diagnosis and treatment of PM, applications of the methods suited to assess prognosis and effectively reduce recurrence and mortality are lacking, therefore, it is urgent to discover non-invasive biomarkers with high sensitivity and specificity to assess the prognosis of PM. In recent years, surgery in combination with chemotherapy and optimization of chemotherapy have improved the survival and life quality of PM patients^23^, despite these improvements, long-term survival in patients with PM is rare, making it difficult to determine which biological factors clearly distinguish between patients with poor and improved OS. To date, pleural biopsy with histopathological evaluation has been shown to be an effective method for PM diagnosis, while the results must be interpreted morphologically, which has some limitations^24^-^26^. Traditional predictive indicators, such as TNM staging and pathological grade, also have a limited ability to predict PM prognosis. As a result, tumor biomarkers are receiving more and more attention due to their less invasive nature. By looking for suitable approaches and using them as entry points between easily detectable biomarkers and patient survival, we can achieve better outcomes from three aspects: disease diagnosis, treatment, and prognosis.

Recent studies have found that miRNAs play critical roles in the occurrence and development of various tumors, and the pivotal function is to regulate gene expression^27^. Interestingly, about 50% of miRNA genes in the human genome are mapped to genomic regions that are highly associated with cancer^28^, ^29^, suggesting their potential in malignancy. In fact, the mechanisms of miRNAs in several malignancies, including PM, have been growing concerns over the past decade, miRNAs were detected in primary tumors and body fluids such as blood, urine and saliva, some studies have also reported their roles in the diagnosis, prognosis and treatment of various cancer types^30^, ^31^. To date, many studies have reported the roles of miRNAs in PM, in particular, some of which highlighted the significance of specific miRNA expression in the prognosis of PM patients^32^.

In our research, we obtained the data of PM samples from TCGA database, univariate Cox regression analysis and multivariate Cox regression analysis illustrated that there were four prognosis-related miRNAs, including hsa-miR-181a-2-3p, hsa-miR-491-5p, hsa-miR-503-5p, and hsa-miR-3934-5p, were identified to establish a 4-miRNA signature, which obtained an AUC value of over 0.8, provided a good predictive ability. Patients were divided into high- and low-risk groups based on the median risk score, we found that the patients in the high-risk group had shorter survival times. In addition, the expression levels of 4 miRNAs showed statistical differences in PM cell lines compared to normal cell line, illustrating that the prognostic model had predictive value. Moreover, the risk score can be used as an independent predictor of PM prognosis.

The roles of the four miRNAs in cancers have been previously explored. Li *et al.* found that miR-181a-2-3p level was notably elevated in human gastric cancer cell lines, facilitated the progression of gastric cancer cell by targeting MYLK, which may be a pivotal prognostic biomarker for gastric cancer^33^, targeting oncogenic miR-181a-2-3p inhibits growth and suppresses cisplatin resistance of gastric cancer^34^. Hsa-miR-181a-2-3p has been reported as a potential prognostic indicator for papillary thyroid cancer, however, its specific functions and molecular mechanisms in papillary thyroid carcinoma have not been elucidated^35^. Downregulation of miR-491-5p induces EMT by regulating SNAIL and FGFR4, thereby promoting gastric cancer metastasis^36^. A miR-491-5p prediction model was developed, the dynamic changes of the miR-491-5p prediction score might be a potential prognostic biomarker for HNSCC^37^, miR-491-5p was downregulated in colorectal cancer tissues and cells, decreased miR-491-5p expression were closely related to differentiation, TNM stage, and poor overall survival^38^. MiR-503-5p inhibited cell epithelial-mesenchymal transitions (EMT) and metastasis by inhibiting WEE1 and predicted the prognosis of hepatocellular carcinoma^39^. Wei *et al.* revealed that downregulation of miR-503-5p contributes to colon cancer tumorigenesis by targeting VEGF-A, modulating the angiogenesis and lymphangiogenesis^40^. Xu and Chen *et al.* observed that p53/miR-503-5p/PUMA signaling axis regulates chemotherapy response in colorectal carcinoma, and suggest that miR-503-5p plays an important role in the development of multidrug-resistance by modulating PUMA expression^41^. Downregulation of miR-3934-5p induces apoptosis and inhibits the proliferation of neuroblastoma cells by targeting TP53INP1^42^, as a tumor suppressor in non-small cell lung cancer, miR-3934-5p can promote the sensitivity of cells to cisplatin by targeting TP53INP1, which is associated with inhibition of cell proliferation and promotion of apoptosis^43^.

To investigate the potential mechanism of four miRNAs in PM progression, bioinformatics analysis was employed to identify target genes, KIF23 and VPS37B were selected as core targets, miRNA regulatory network showed interactions between miRNAs and target genes, suggesting complex mechanisms among them. Further functional analysis showed that miRNA regulatory network genes may participate in the occurrence and development of PM through multiple tumor-associated pathways, including PI3K/Akt signaling pathway, p53 signaling pathway, and Wnt signaling pathway, etc. We found that KIF23 and VPS37B expressions were closely correlated with several immune cell infiltrations, infiltration levels of these cells had a significant impact on survival outcome. The KIF23 gene encodes a member of kinesin protein involved in regulating cytokinesis^44^, its inhibiting suppresses midbody formation, hence the completion of cytokinesis hampering cancer cells proliferation *in vitro* and *in vivo*^45^, ^46^. Overexpression of KIF23 was recently confirmed in a tissue microarray of 53 mesothelioma samples, and a shorter overall survival was observed in patients who received curative resection with tumors displaying high KIF23 expression (*p*=0.0194)^47^, suggesting its value as a potential prognostic marker. The VPS37B gene is a component of the ESCRT-I complex, as well as a regulator of vesicular trafficking process. Kolmus *et al.* revealed that VPS37B was decreased in the advanced stages of CRC^48^. Currently, researches on mechanism of VPS37B in relation to tumors are limited, deserving further investigation. Therefore, we speculate that these genes may be involved in the regulation of tumor microenvironment via affecting immune infiltration, further undermining the prognosis of PM patients.

The specific linkages between the two key targets and immune cell populations were further analyzed. We observed that activated NK cells in VPS37B high expression group were significantly decreased, suggesting that high expression level of VPS37B may be related to the decrease in the number of NK cells as well as dysfunction, resulting in tumor recurrence and metastasis. NK cell decrease is also the reason for the poor prognosis of conventional treatments (surgery, radiotherapy, chemotherapy, etc.), which may also be one of the reasons for PM growth, proliferation, and infiltration. In clinical studies, low NK cell activity has been observed to be associated with increased risk of cancer susceptibility and metastasis^49^-^51^. NK cells can also drive tumor inflammation by producing chemokines (XCL1 or XCL2) or cytokines (e.g., IFN-γ) and recruiting and maturing other immune cells to form the tumor microenvironment^52^-^54^. Therefore, immune intervention strategies targeting NK cells have great potential in the immunotherapy of PM.

In summary, our study constructed a miRNA prognostic risk model which can effectively distinguish between high- and low-risk groups of PM patients and has good sensitivity and specificity for evaluating the prognosis of PM patients, it can be used as a biomarker for predicting the prognosis of PM. By comprehensively analyzing multiple bioinformatics public databases, we further explored their potential biological functions in the occurrence and development of PM, but there are still certain limitations in our study, firstly, patients at early-stage can benefit greatly from clinical intervention, this model cannot effectively distinguish high-risk patients in early stage of PM, so the model cannot provide a reference for more effective clinical interventions, such as taking a more thorough treatment approach, shortening the follow-up observation interval, etc. Secondly, this model was only validated in the TCGA database, lacking of external independent datasets, makes it hard to further validate the effectiveness of the model. Thirdly, establishment of miRNA prognostic models specific to different pathological subtypes of PM still needs further exploration, which needs to be improved in subsequent studies.

## Supplementary Material

Supplementary figure.

## Figures and Tables

**Table 1:** Univariate Cox regression analysis results.

miRNA	HR	HR.95L	HR.95H	*p* -value
hsa-miR-3934-5p	0.530250	0.375416	0.748941	0.000317
hsa-miR-3129-3p	1.713393	1.235157	2.376795	0.001261
hsa-miR-181a-2-3p	2.159816	1.451902	3.212892	0.000145
hsa-miR-29c-3p	0.591147	0.427284	0.817852	0.001504
hsa-miR-503-5p	1.571507	1.171911	2.107356	0.002530
hsa-miR-491-5p	0.628657	0.467881	0.844680	0.002070
hsa-miR-203b-3p	0.811773	0.696803	0.945713	0.007444

Note: HR represents hazard ratio. HR.95L represents low 95% confidence interval of HR. HR.95H represents high 95% confidence interval of HR.

**Table 2:** Multivariate Cox regression analysis results.

miRNA	Coef	HR	HR.95L	HR.95H	*p* -value
hsa-miR-3934-5p	-0.38379	0.681271	0.460666	1.007521	0.054551
hsa-miR-181a-2-3p	0.563118	1.756140	1.103044	2.795924	0.017630
hsa-miR-503-5p	0.329360	1.390078	1.043388	1.851963	0.024440
hsa-miR-491-5p	-0.49509	0.609513	0.440836	0.842732	0.002744

Note: Coef represents regression coefficient of each miRNA. HR represents hazard ratio. HR.95L represents low 95% confidence interval of HR. HR.95H represents high 95% confidence interval of HR.
