# Supplementary figures and images for: Establishment and bioinformatics analysis of a four-miRNA prognostic signature for pleural mesothelioma
Source: J Cancer. 2024 Oct 21;15(20):6505–20. doi: 10.7150/jca.101914 (PMC11632982; doi:10.7150/jca.101914)

**A**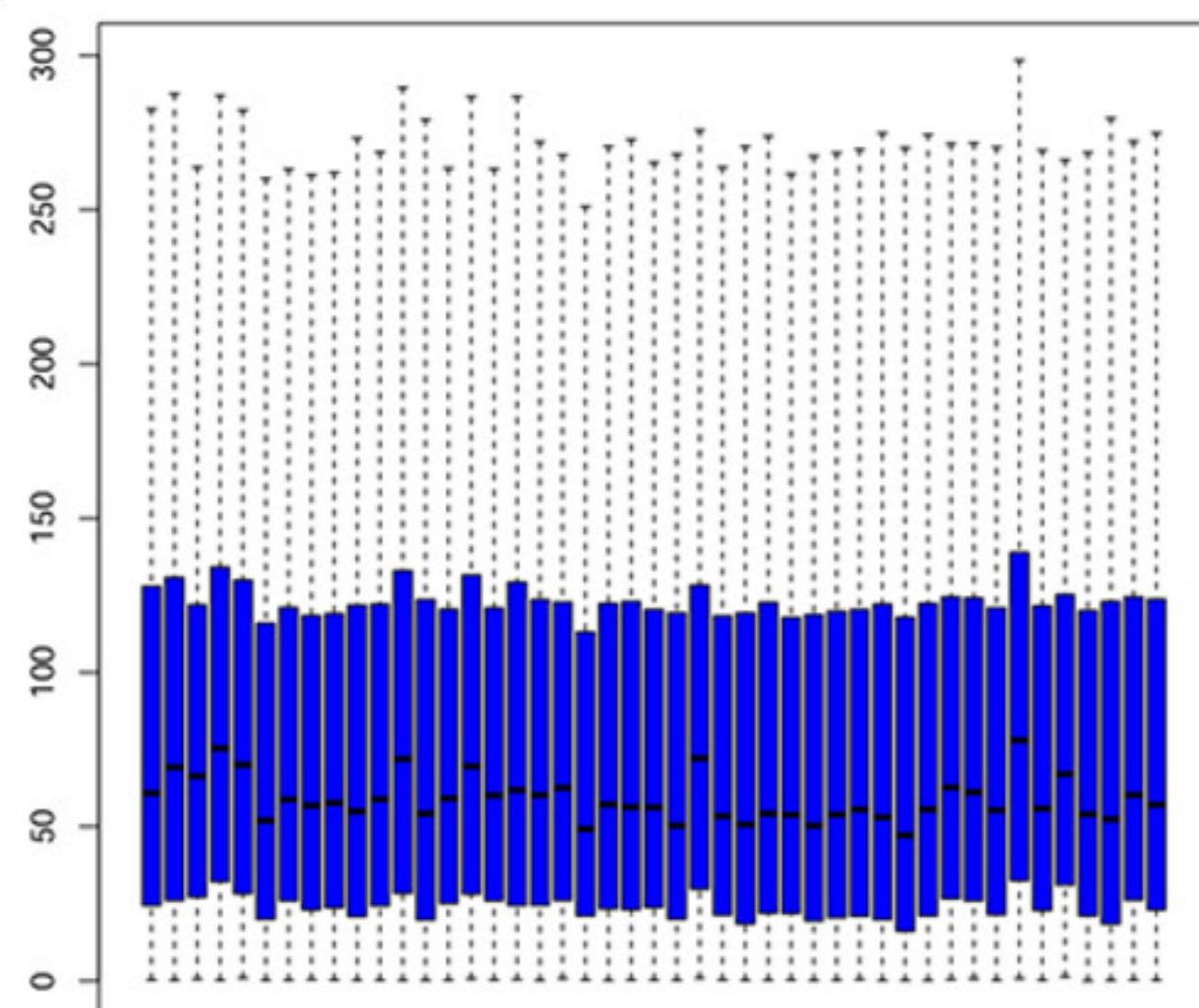**B**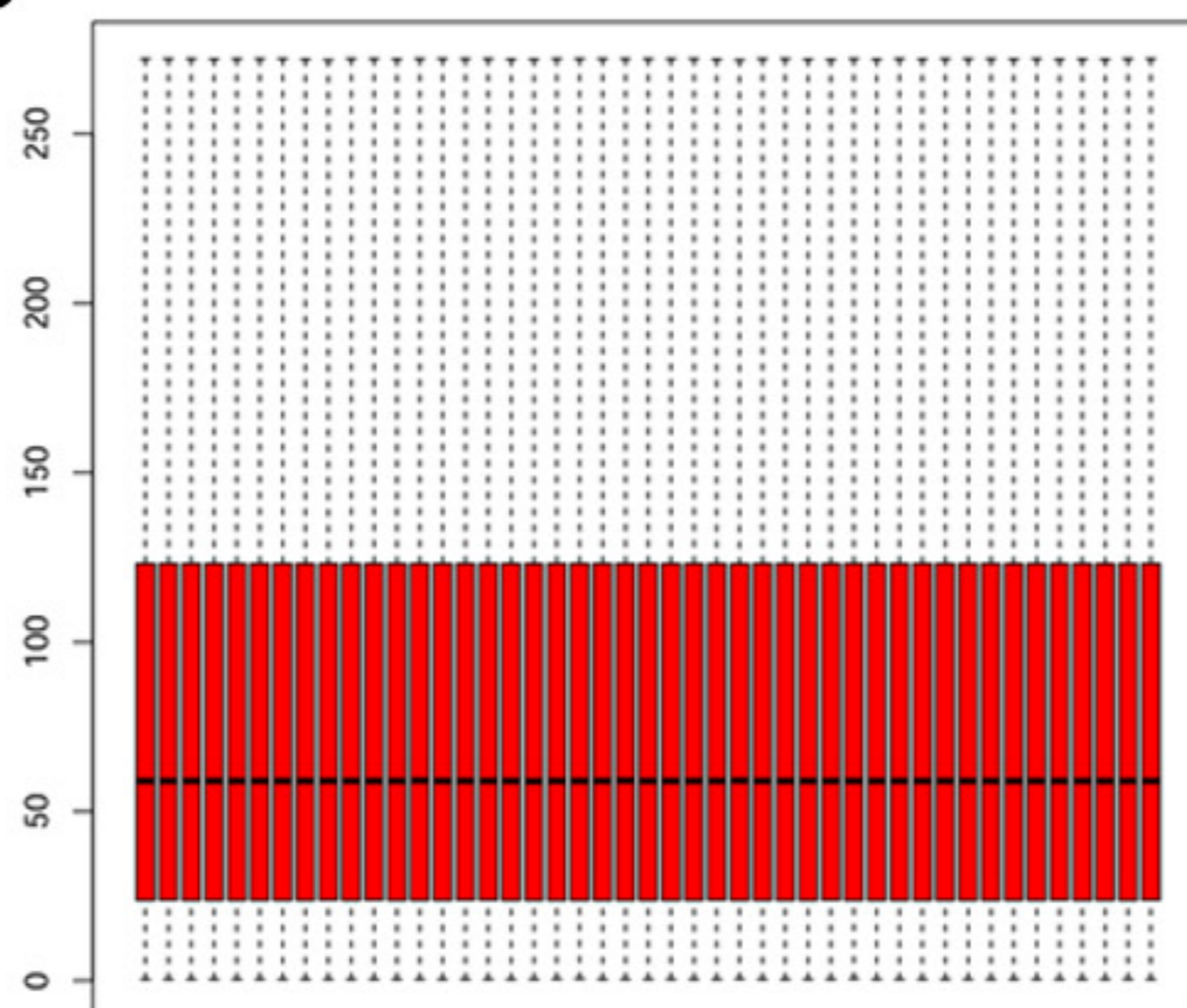**C**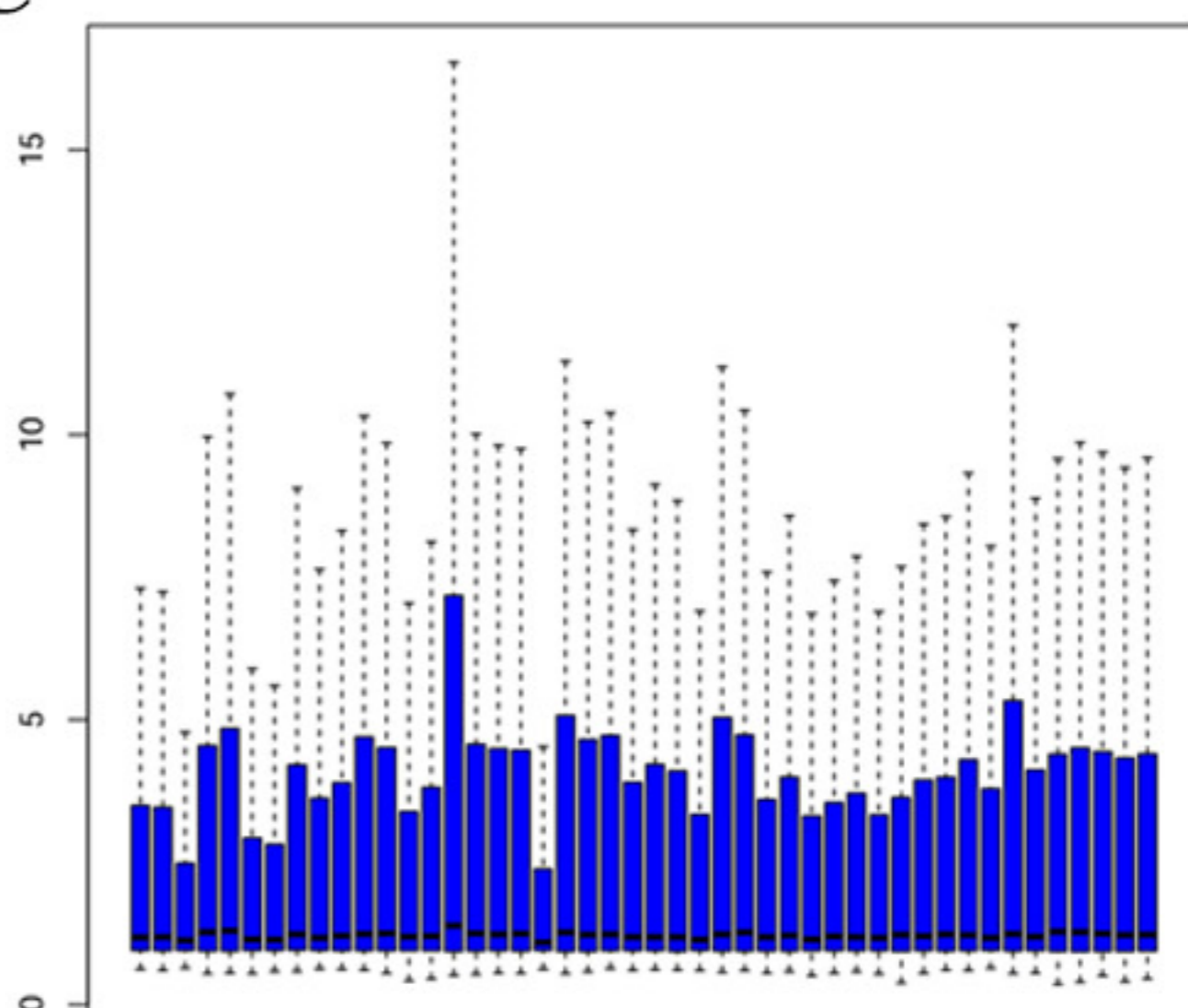**D**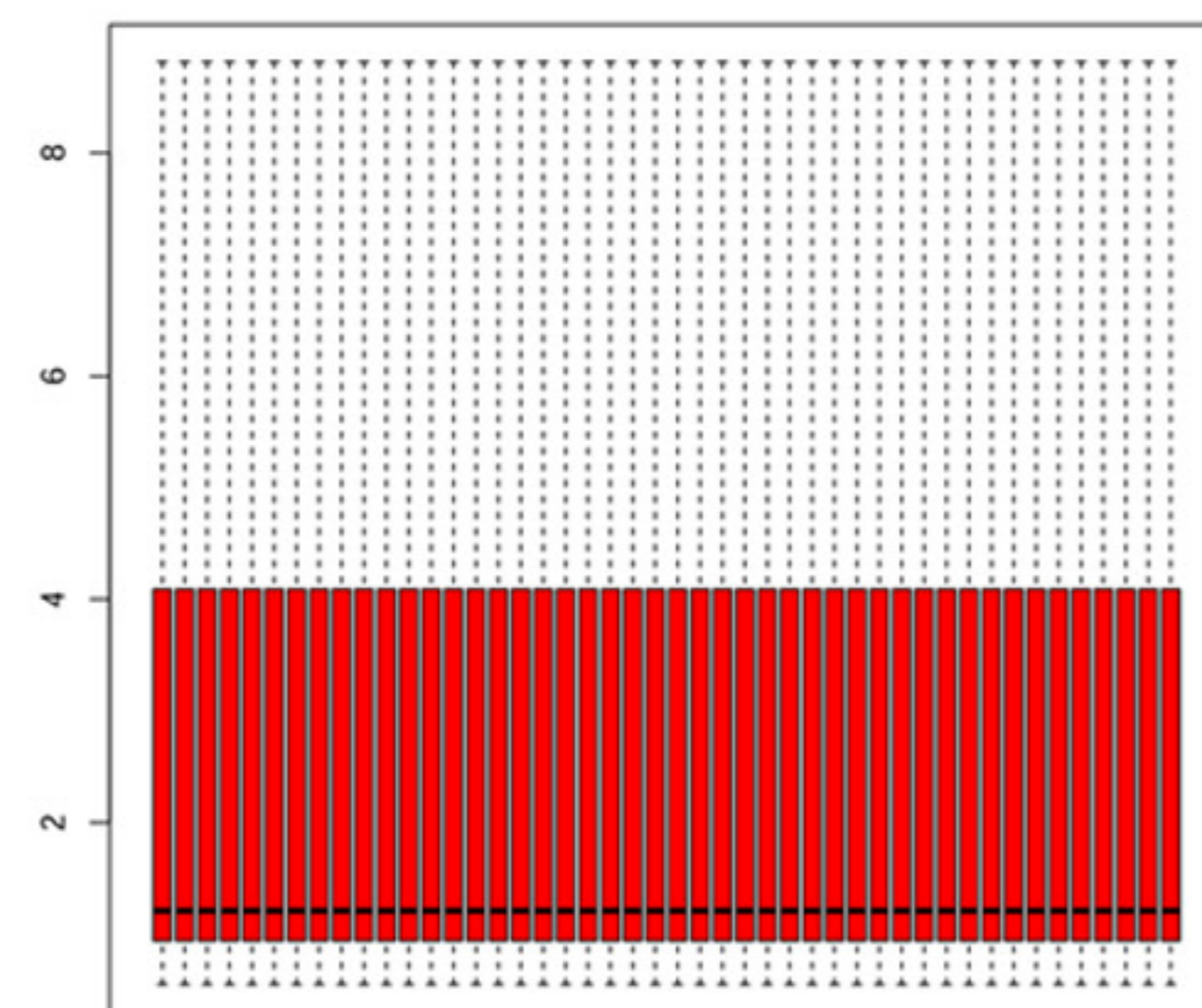**E**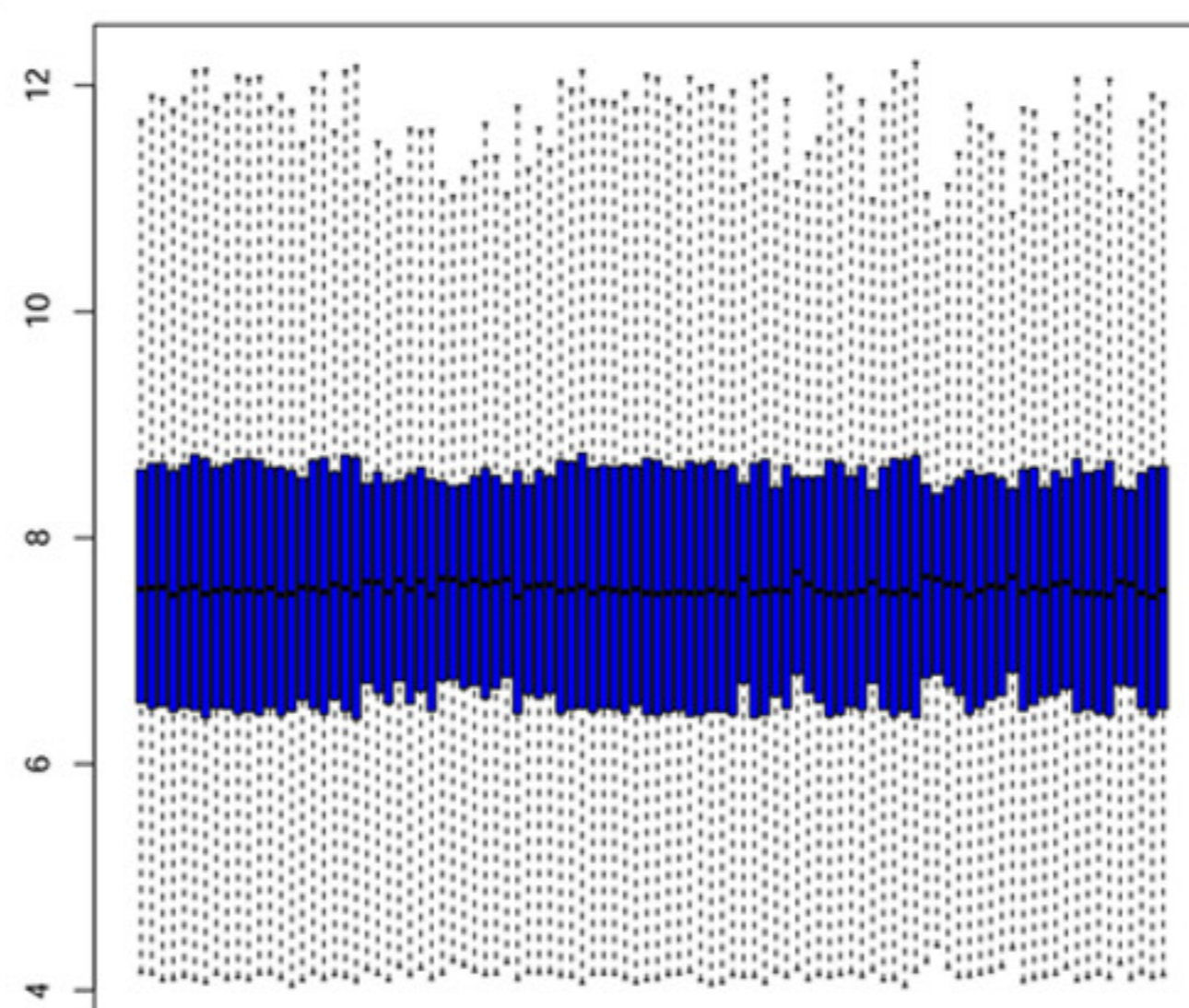**F**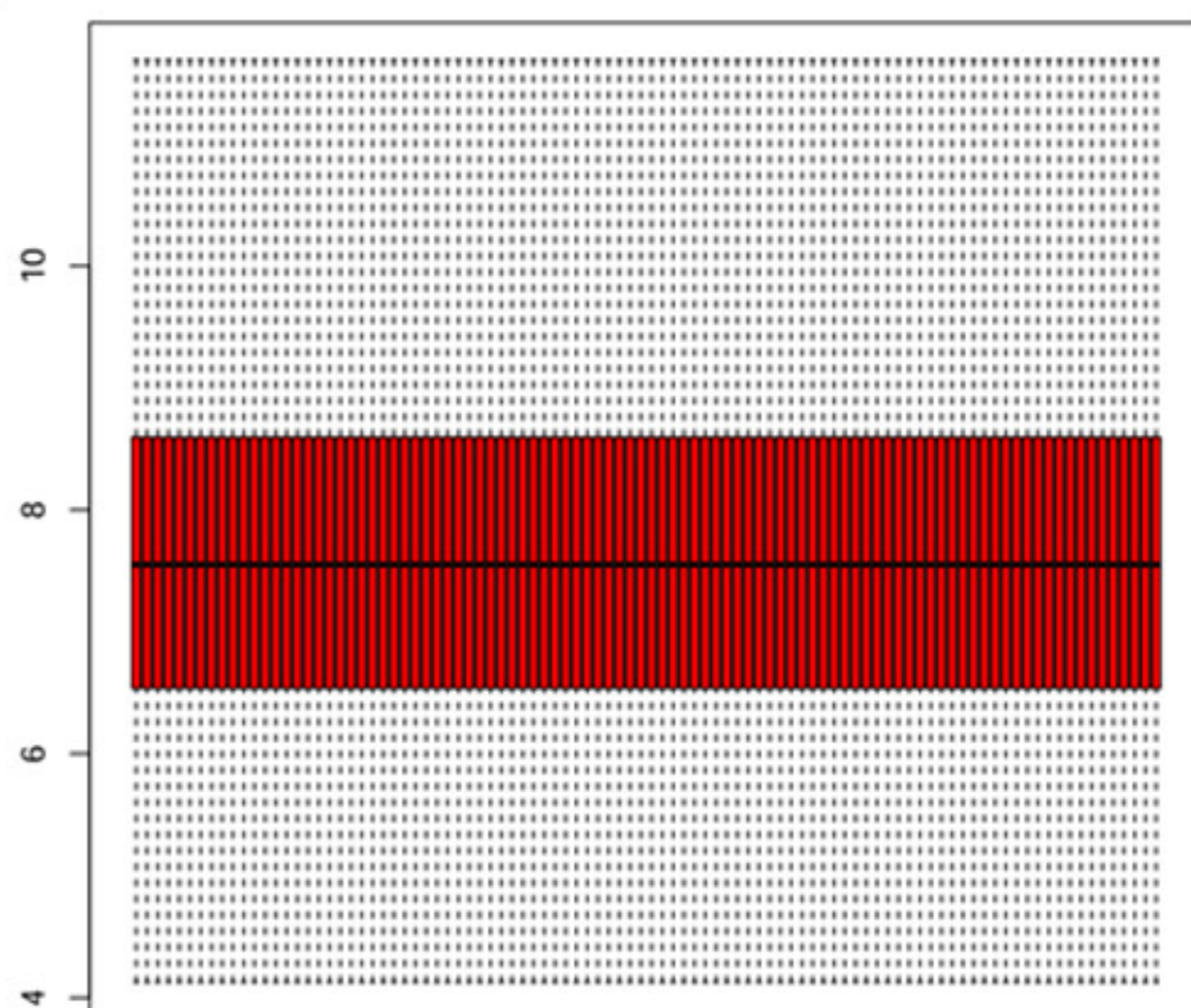**G**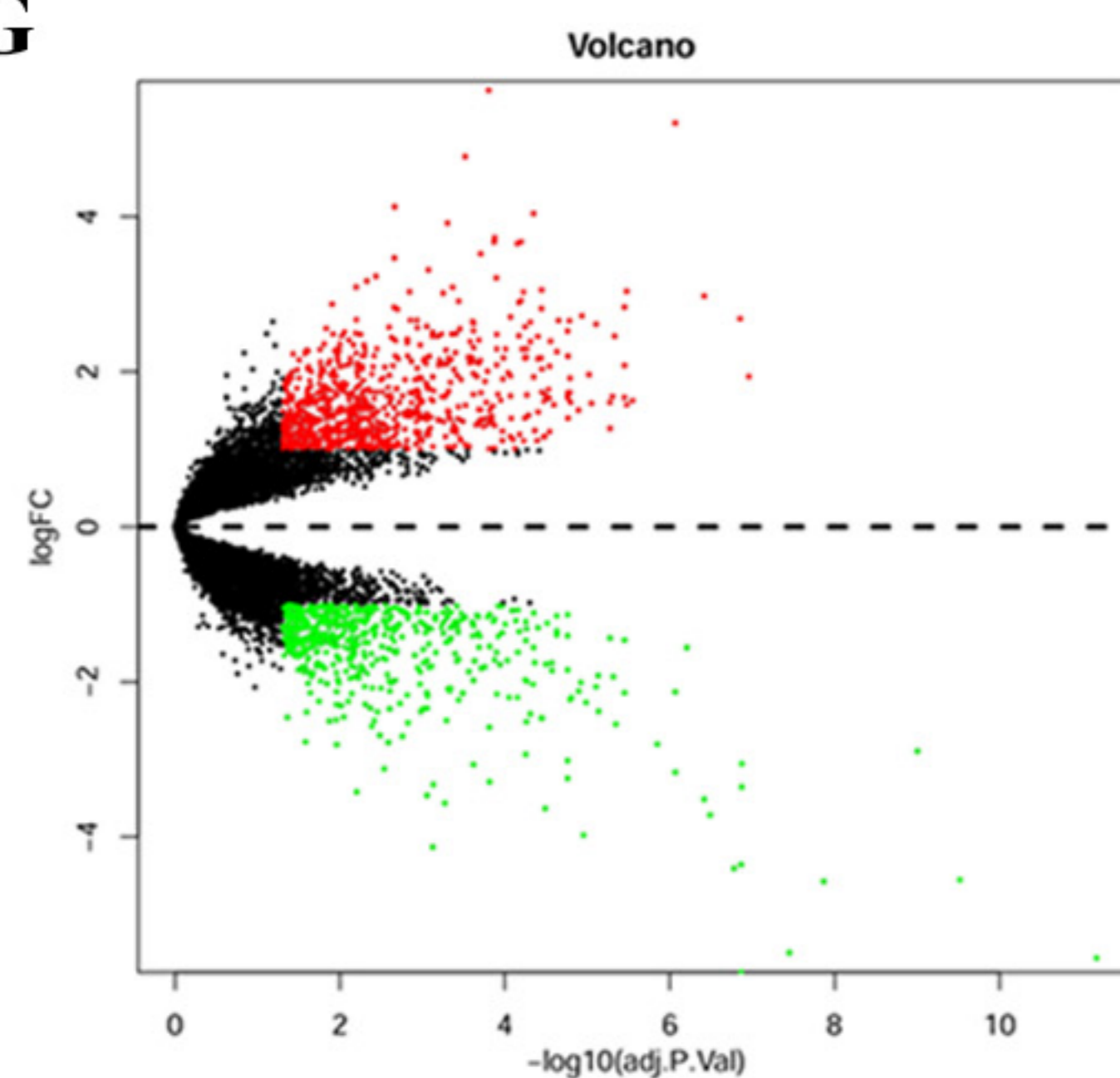**H**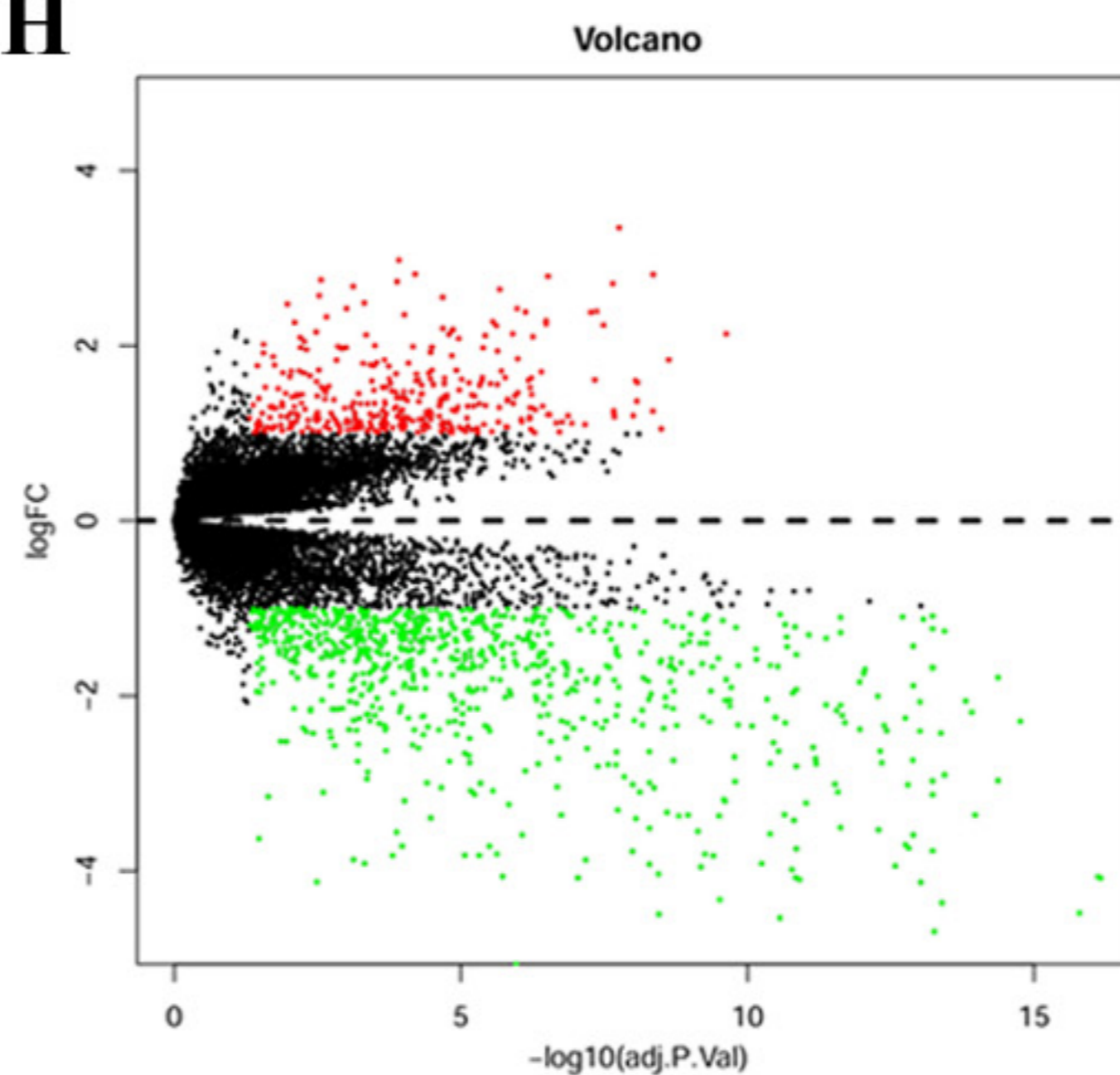**I**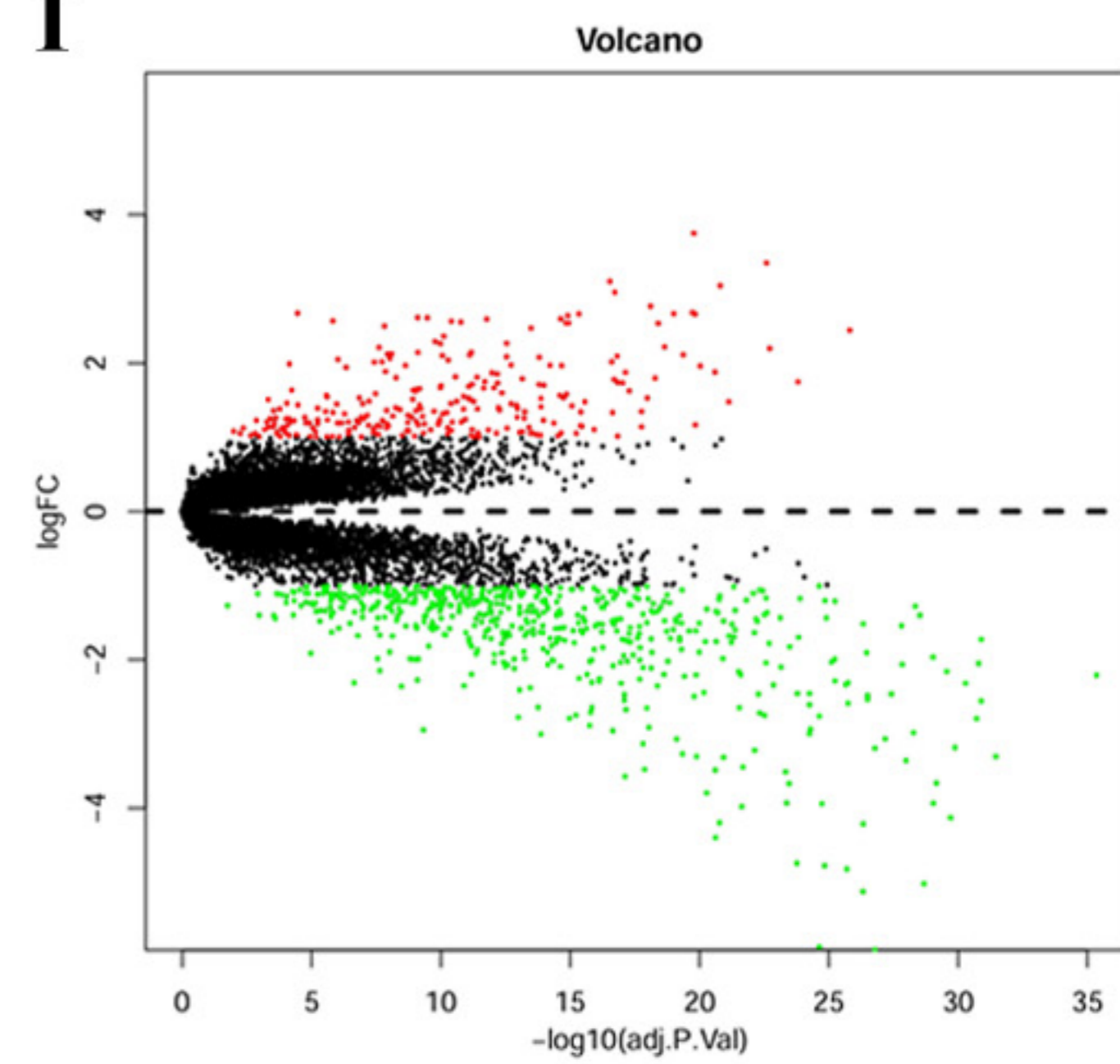**J**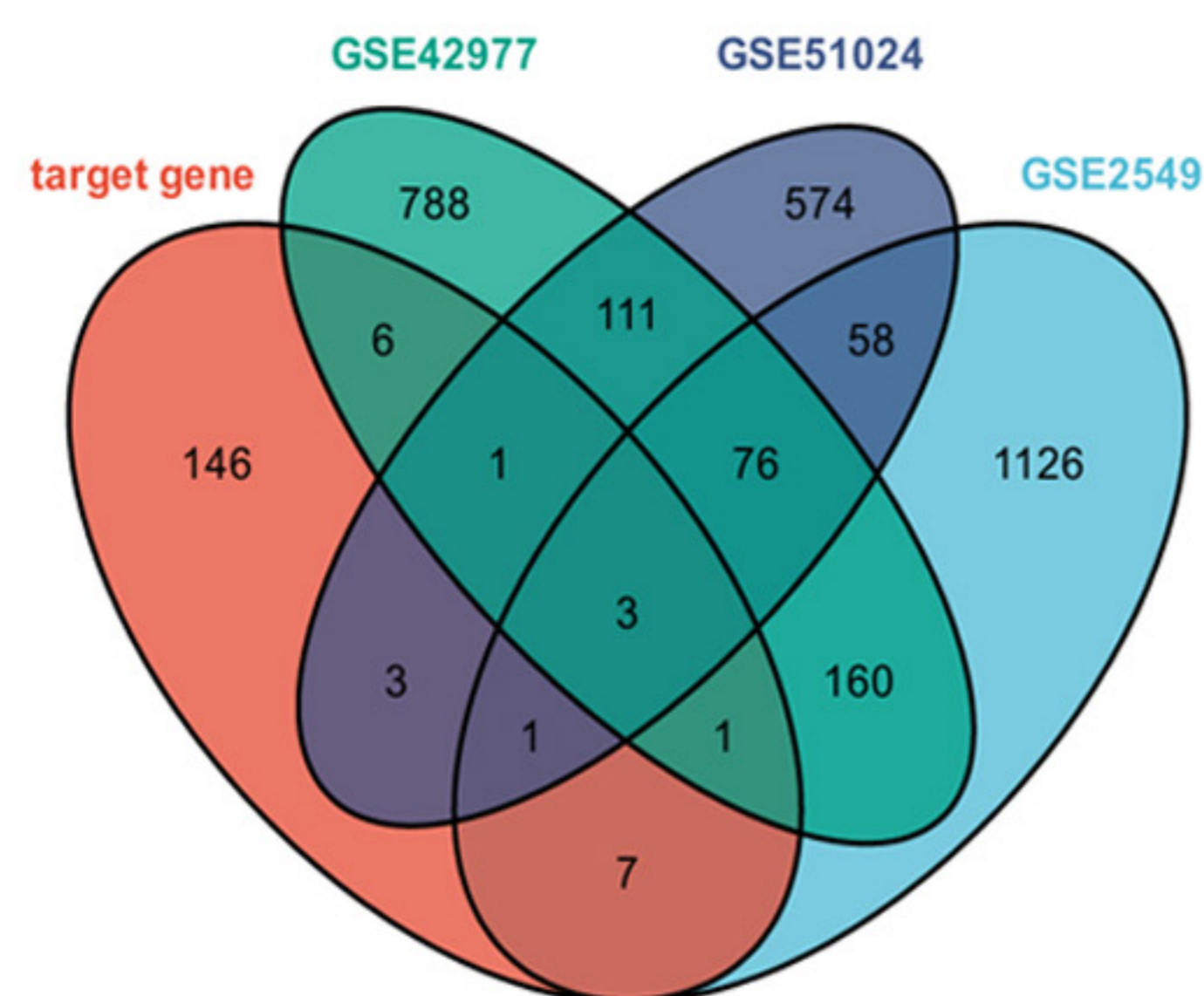**K**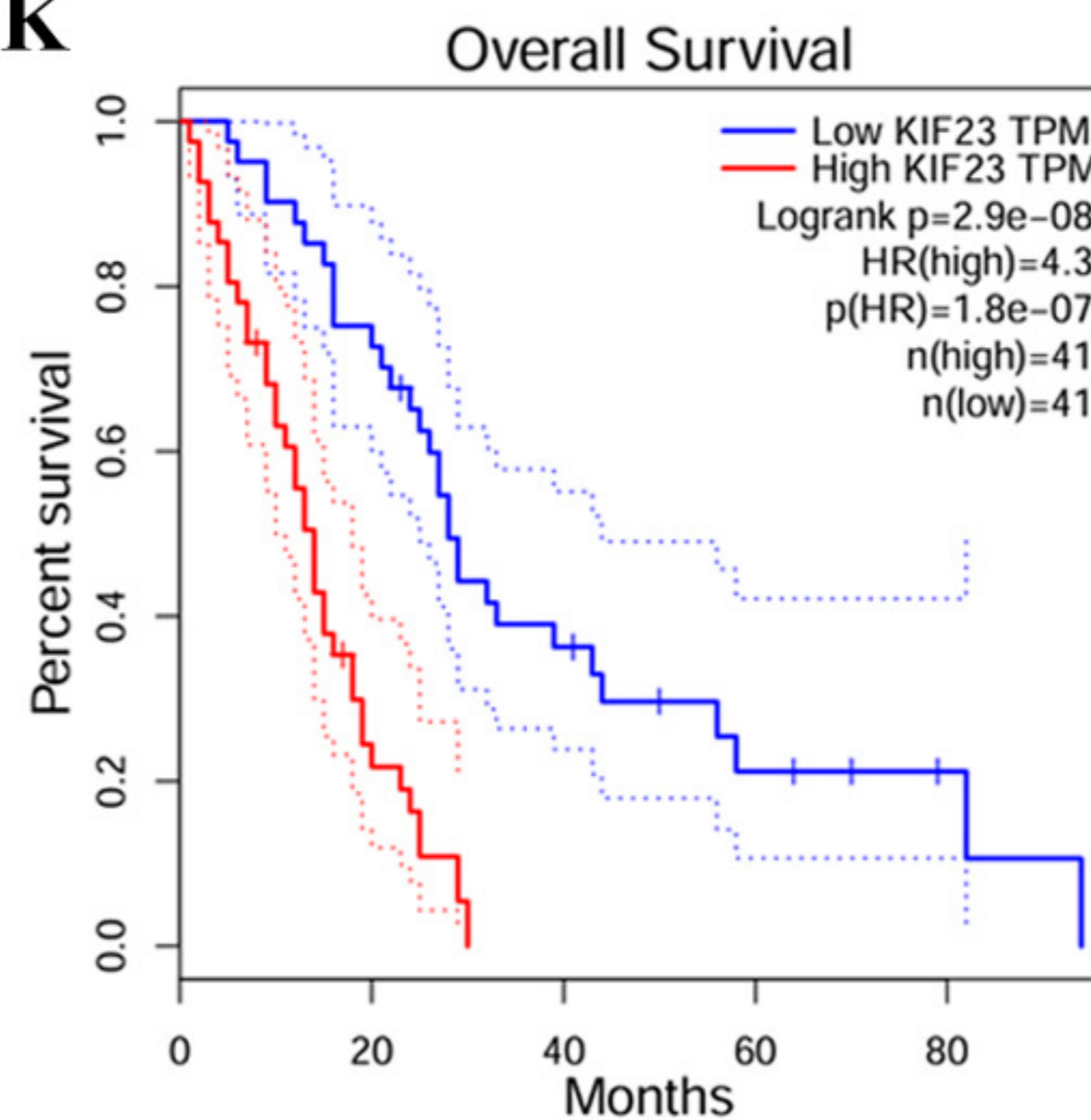**L**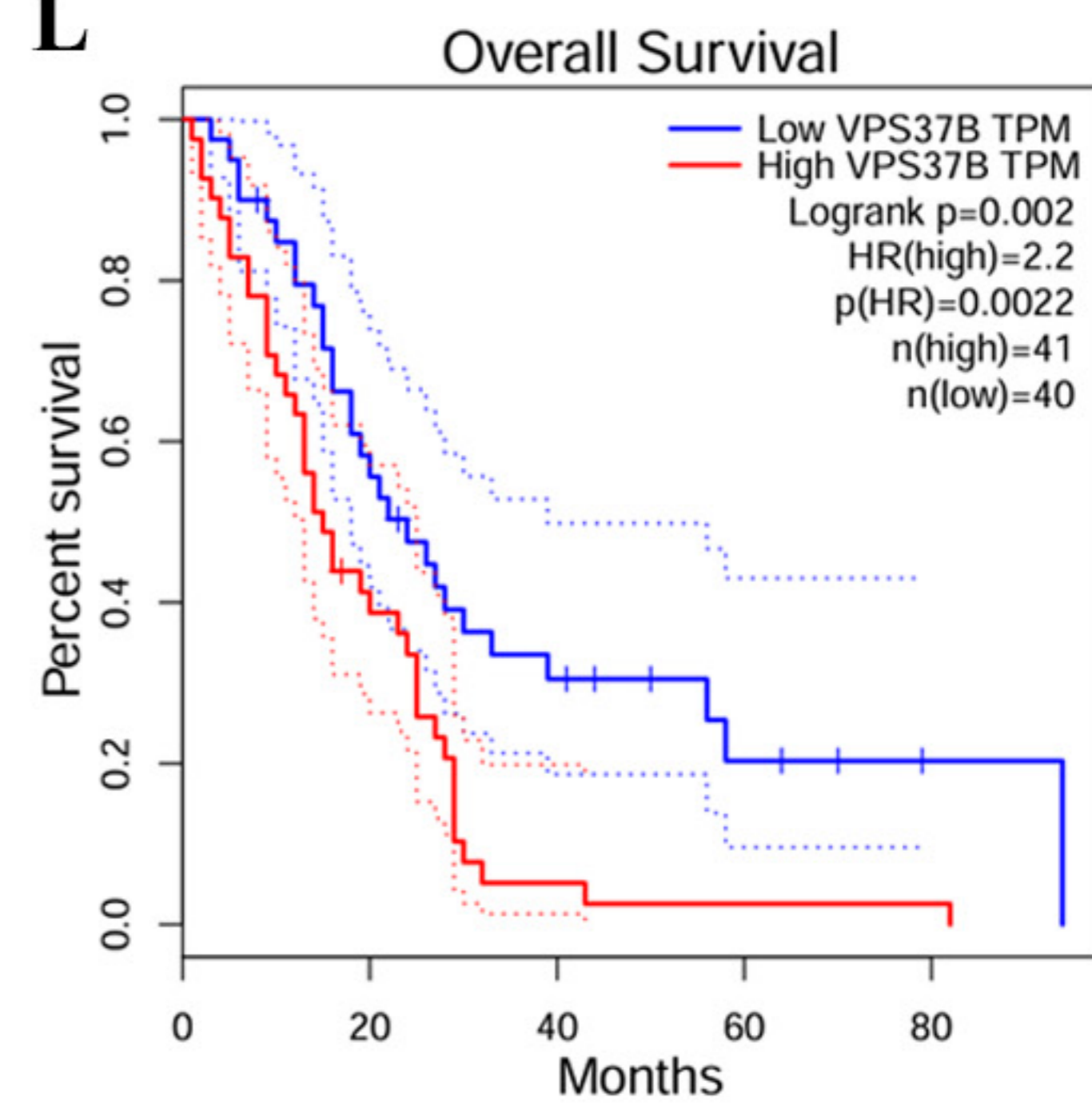

Supplement: Supplementary file 1 — Supplementary figure. [file jcav15p6505s1.pdf]
